# Spatial and Phenotypic Heterogeneity of ILC Subsets in Mouse Lung Under Type 2 Inflammatory Conditions

**DOI:** 10.1002/eji.70223

**Published:** 2026-06-17

**Authors:** Sandy Kroh, Anna Pascual‐Reguant, Artür Manukyan, Ralf Uecker, Robert Günther, Lars Philipsen, Ralf Koehler, Peggy Mex, Markus Landthaler, Raluca A. Niesner, Anja E. Hauser

**Affiliations:** ^1^ Department of Rheumatology and Clinical Immunology Charité‐Universitätsmedizin Berlin, Corporate Member of Freie Universität Berlin and Humboldt‐Universität zu Berlin Berlin Germany; ^2^ Immune Dynamics Deutsches Rheuma‐Forschungszentrum (DRFZ) a Leibniz Institute Berlin Germany; ^3^ Centre Nacional d'Anàlisi Genòmica (CNAG) Barcelona Spain; ^4^ Universitat de Barcelona Barcelona Spain; ^5^ Berlin Institute for Medical Systems Biology (BIMSB) Max Delbrück Center for Molecular Medicine Berlin Germany; ^6^ Health Campus Immunology Infectiology and Inflammation Otto‐von‐Guericke‐University Magdeburg Germany; ^7^ Multi‐parametric bioimaging and cytometry (MPBIC) core facility University Hospital Otto‐von‐Guericke‐University Magdeburg Germany; ^8^ Institute of Cellular and Molecular Immunology University Hospital Otto‐von‐Guericke‐University Magdeburg Germany; ^9^ EMBL Node in Single Molecule Science Department of Molecular Medicine School of Biomedical Sciences Faculty of Medicine & Health University of New South Wales Sydney Australia; ^10^ Veterinary Medicine Dynamic and functional in vivo Imaging Freie Universität Berlin Berlin Germany; ^11^ Biophysical Analytics Deutsches Rheuma‐Forschungszentrum (DRFZ) a Leibniz Institute Berlin Germany; ^12^ Institut für Biologie Humboldt‐Universität zu Berlin Berlin Germany

**Keywords:** IL‐33, innate lymphoid cells, mucosal immunology, proteomics, systemic inflammation model

## Abstract

As key regulators of mucosal immunity, innate lymphoid cells (ILCs) are involved in tissue homeostasis, inflammation, and repair. Studying ILCs within their native microenvironment remains challenging due to the low abundance of these tissue‐resident immune cells. Here, we applied cyclic multiplex immunofluorescence, namely multiepitope ligand cartography (MELC), in a systemic IL‐33‐induced type 2 inflammation model to spatio‐temporally characterize ILC phenotype and localization in mouse lungs. Niche analysis with all identified cell types resulted in four distinct niches and an expansion of a mixed B and Plasma cell (BPC)/blood endothelial cell (BEC) niche, while the niche predominated by blood endothelial cells decreased at IL‐33 day 3. Spatial neighborhood and coenrichment analyses revealed ILC2 accumulation in myeloid‐rich peri‐lymphatic niches at early time points of IL‐33‐mediated inflammation. ILC2s were in direct contact with activated alveolar macrophages and lymphatics. While they expressed ICOS under homeostatic conditions, pronounced expression of MHCII at days 1 and 3 of IL‐33 stimulation was observed. Unlike ILC2s, NK cells/ILC1s were coenriched near blood vessels, next to B cells and plasma cells. Our findings demonstrate the utility of spatial multiplex imaging for dissecting rare immune cell localization and phenotypes and uncover dynamic, tissue‐specific remodeling of ILC niches during early type 2 inflammation.

## Graphical abstract

Using cyclic multiplex microscopy in an IL‐33‐driven type 2 inflammation model, we validated a spatial workflow mapping rare lung ILCs. High‐dimensional analysis revealed a conserved peri‐lymphatic niche at early time points of inflammation where ILC2s and myeloid cells co‐localize with LYVE1+ vessels, highlighting a structured immunological microenvironment across inflammatory states.

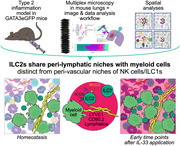

**Abbreviations**
AL: annotation level
AREG: amphiregulin
BEC: blood endothelial cells
BPC: B and plasma cells
CCL: C–C motif chemokine ligand
CCR: C–C motif chemokine receptor
CD: cluster of differentiation
DAPI: 4',6‐diamidino‐2‐phenylindole
EMCN: endomucin
EOMES: eomesodermin
FN: fibronectin
FOV: field of view, tissue region
GATA3: GATA binding protein 3
ICOS: inducible T cell co‐stimulator
IFNγ: interferon‐γ
IL: interleukin
ILC: innate lymphoid cell
KLRG1: killer cell lectin‐like receptor G1
LYVE1: lymphatic vessel endothelial hyaluronan receptor 1
MELC: multiepitope ligand cartography
MHC: major histocompatibility complex
NK cell: natural killer cell
PDGFRα: platelet‐derived growth factor receptor‐α
PDPN: podoplanin
ROI: region of interest
RORγt: RAR‐Related Orphan Receptor C
Sca1: stem cells antigen‐1
TBET: T‐box expressed in T cells
Th cell: T helper cell
TNF‐α: tumor necrosis factor‐α
Treg cell: regulatory T cell
VIM: vimentin

## 1 Introduction

Innate lymphoid cells (ILCs) are key regulators of inflammation, tissue repair, and immune coordination [1, 2, 3] and are generally known as the innate counterpart of T cell subsets sharing transcriptional, phenotypical, and functional similarities [4]. However, they do not have antigen‐specific receptors, but they sense the presence and react upon signals mediated via cytokines, metabolites, and direct interactions with cellular neighbors in their microenvironment [5, 6, 7, 8, 9]. Various ILC subtypes have been reported: Type 1 ILCs (ILC1) are marked by expression of the master transcription factor TBET, and they secrete IFN‐γ and TNFα in the context of intracellular viral and bacterial infections as well as cancer [4]. NK cells partly share their marker, cytokine, and transcriptional profiles with ILC1s, but represent a cytotoxic subset, mirroring the adaptive branch of CD8^+ ^T cells. Like ILC1s, NK cells are involved in intracellular defense in the context of type 1 immunity and are potent producers of IFN‐γ and TNF‐α [10, 11, 12]. Associated with type 2 immunity, ILC2s mirror T helper (Th)2 cells through the expression of high levels of the master transcription factor GATA3 and secretion of type 2 cytokines such as IL‐5, IL‐9, and IL‐13 during helminth infection [13]. Furthermore, allergic processes such as asthma are associated with ILC2s, as well as chronic fibrotic diseases [14, 15, 16, 17]. The master transcription factor RORγt defines ILC3s, which are potent producers of barrier‐associated cytokines such as IL‐17A, IL‐17F, and IL‐22. ILC3s are mainly known for their role in type 3 inflammatory processes, such as extracellular pathogens, as well as maintaining barrier integrity [18]. The ILC subgroups represent heterogeneous populations with tissue‐ and environment‐specific variations and adaptations. ILCs possess the capacity to change their identity, a process known as plasticity [19, 20, 21, 22, 23].

ILCs are extremely rare, raising the question of how such scarce cells exert disproportionate immunological influence. One possible answer lies in their spatial organization: their function may depend not only on their presence but on where they are located within the tissue and whom they interact with. ILCs are potent sensors of changes in their microenvironment; conversely, they are able to shape their surroundings by secreting cytokines. Human ILCs localize in fibrovascular niches that are conserved throughout different inflamed tissues such as the tonsil and intestines [24]. This aligns with publications showing mouse ILC2s to localize in adventitial cuffs together with adventitial stromal cells, dendritic cells (DCs), and T regulatory (Treg) cells in various tissues [25]. It highlights how cells are not evenly distributed within the tissue but instead accumulate in micro‐anatomical sites together with specific cell types. Those functional hubs are shaped to support cellular functions such as local inflammation and are called niches. Dissociative methods like flow cytometry and single‐cell sequencing have been widely used to study ILCs in recent years; however, they lack spatial resolution.

While spatial technologies are frequently used to study the tumor niche [26, 27, 28], other fields in immunology have only started to take advantage of spatial information recently [29, 30, 31, 32]. Limitations in imaging technologies and analysis pipelines have hindered the study of rare immune populations like ILCs in situ. Current knowledge of the spatial distribution of ILCs in mice has used low‐plex immunofluorescence technologies, ligand‐receptor analysis approaches in single‐cell data, or other functional assays [33, 34, 35, 36, 37]. Those approaches only enable the visualization of either a low number of markers and, accordingly, cell types, or represent an indirect approach, missing actual spatial data. An in‐depth analysis of ILCs in multiple organs, achieving single‐cell resolution and the separation of nine different cell types, gaining valuable information about human tonsil ILCs localizing together with plasma cells in fibrovascular niches, has been performed [24]. The formation of localized ILC microenvironments in the context of inflammation has received comparatively little attention [24, 32, 38]. These inflammation‐induced changes are especially relevant at barrier surfaces such as the lungs, where immune responses must simultaneously ensure protection against pathogens and maintain tissue homeostasis. Although mainly described as tissue‐resident cells, inflammatory stimuli can induce inter‐organ migration of activated ILC2s from the gut and ILC2 progenitors from the bone marrow toward the lung [39, 40, 41].

Here, we established the multiplex immunofluorescence technology multiepitope ligand cartography (MELC) [42] in mouse lung samples and combined it with an IL‐33 systemic inflammation model to spatially profile multiple immune cell types—including rare subsets such as ILCs—and their adaptations during a type 2 inflammation at single‐cell resolution. IL‐33 is a cytokine associated with type 2 inflammation that is released upon tissue destruction, injury, or uncontrolled cell death. IL‐33 is also associated with lung fibrosis and triggers a quick activation and expansion of ILC2s in various organs [43]. Most studies investigating ILCs in the context of IL‐33–induced inflammation have concentrated on later stages of the immune response [39, 44, 45]. As a result, little is known about the spatial organization and phenotypic changes of ILCs during the early phase of inflammation—particularly within the first three days. Yet ILCs are known for their ability to respond rapidly to alarmins such as IL‐33 and for their strategic localization within barrier tissues. By analyzing tissue at various time points after systemic IL‐33 application, we are able to characterize ILC subsets, define their spatial niches, and characterize their spatial adaptations during the earliest stages of type 2 inflammation. This approach represents a systematic dissection of cellular interactions in tissue‐specific niches and offers a powerful tool for studying the spatial logic of mucosal immunity.

## 2 Results

### 2.1 Analyzing Systemic Inflammation With Cyclic IF to Study ILC Niches in Mouse Lungs

**FIGURE 1:**
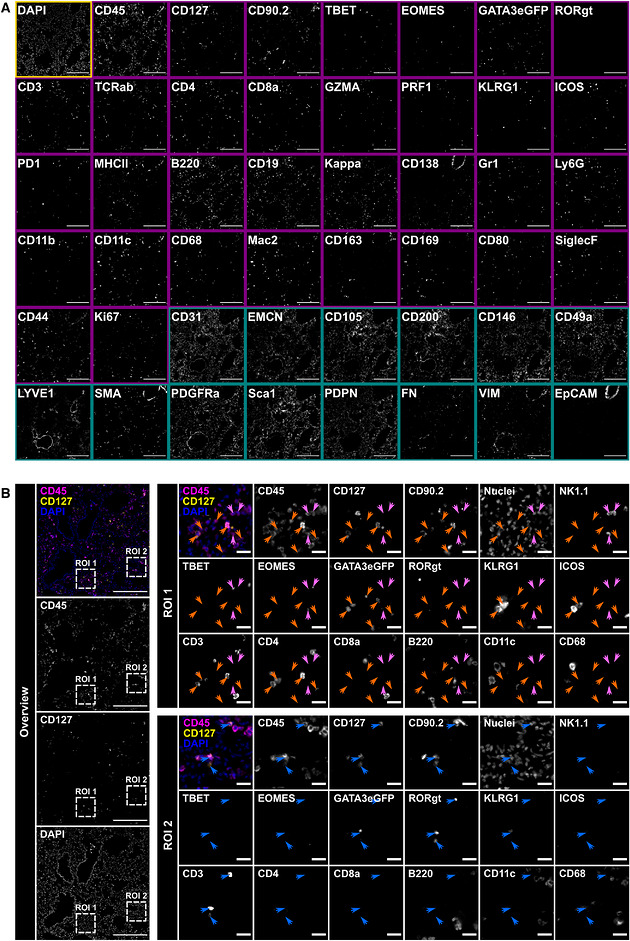
Overview of the established MELC panel in mouse lungs. (A) An established 48‐marker panel acquired in an exemplary tissue region of mouse lungs. Each image shows the same tissue region stained for diverse immune and functional markers (purple), and structural markers (cyan), as well as nuclei stain (Yellow). Scale bars represent 200 µm. EMCN: endomucin; FN: fibronectin; GZMA: granzyme A; PDPN: podoplanin; PRF1: perforin; SMA: smooth muscle actin, VIM: vimentin. (B) IF overlay of CD45 (magenta), CD127 (yellow), and DAPI (blue) of a representative region of mouse lungs. Boxes mark two zoomed‐in ROIs showing additional greyscale single marker images of additional markers. GATA3eGFP^+^ cells (orange), TBET and/or EOMES‐expressing cells (pink), and RORγt^+^ cells (blue) are marked by arrowheads. Scale bars in overview represent 200 µm; scale bars in ROIs represent 20 µm. ROI: region of interest.

First, we designed an antibody panel for MELC with the aim of identifying ILCs, their subtypes, as well as other immune and nonimmune cell types that would enable subsequent spatial neighborhood analysis of ILCs (Figure 1A). As the reliability of markers used for labeling represents the basis for all subsequent analyses, we validated the stainings by overlaying various marker combinations and assessed expected co‐expression patterns of different cell types, including lymphocytes, myeloid cells, as well as endothelial and epithelial cells (Figure 1B; Figure S1A–C). As general inclusion markers for ILCs, we used CD127 and CD90.2. The ILC signature transcription factors TBET, GATA3, and RORγt, as well as the NK cell transcription factor EOMES, were included to differentiate between ILC1s, ILC2s, ILC3s, and NK cells, respectively [4]. We confirmed the expected nuclear location of the transcription factors and found localization within the cytoplasmic/membrane signal of other ILC inclusion markers, such as CD127 and CD90.2. Cells that expressed GATA3, RORγt, TBET, and EOMES partly co‐expressed CD3 (Figure 1B), indicating the presence of T helper subtypes Th1, Th2, and Th17. Although we tested various GATA3 antibodies, the resulting staining was either weak or unreliable. ILC2s are described as the predominant ILC subtype in mouse lung [46]; therefore, we decided to take advantage of a GATA3eGFP reporter mouse strain. Combining the GATA3eGFP reporter with an anti‐GFP antibody resulted in strong staining of GATA3 in mouse lung tissues (Figure 1A) and co‐expression of GATA3eGFP with ICOS and KLRG1 (Figure 1B; orange arrow heads), confirming ILC2 and Th2 identity. Additionally, we established other markers that have been previously described in the context of ILC biology [47], as well as markers for immune and nonimmune cells, summarized in Table S1. We confirmed co‐expression of markers that were expected in different T cell subtypes (CD3, CD4, CD8a; Figure S1A, blue and green arrow heads), cells of the B lineage (B220, kappa light chain, IRF4; Figure S1A, pink arrow heads), myeloid cells (CD11c, CD68; Figure S1A, orange arrow heads), as well as endothelial cells (CD31, endomucin [EMCN], LYVE1, CD90.2, CD200, CD146, CD105; Figure S1B) and epithelial cells (EpCAM, CD44, CD138; Figure S1C). Nuclear markers were included to evaluate tissue quality and integrity in each experiment.

ILCs only constitute around 0.15% to 1% of the immune cell compartment in the mouse lung, as shown by flow cytometry [48]. To investigate how a potential ILC niche is influenced by inflammatory stimuli, we decided to analyze a systemic inflammation model based on consecutive IL‐33 i.p. injections that have been described to trigger a strong type 2 response in various organs and activate ST2^+^ ILC2s [44, 45, 49, 50, 51, 52]. In preliminary experiments, we acquired a MELC dataset of mouse lung samples 3, 6, and 28 days after IL‐33 application and saw a significant increase in ILC2s at day 3 of IL‐33 application (data not shown). Therefore, we decided to focus on very early time points of inflammation, namely day 1, 2, and 3 after IL‐33 injection. Twelve‐ to fourteen‐week‐old females of GATA3eGFP reporter mice were i.p.‐injected with 300 ng IL‐33 on up to three consecutive days, organs were isolated 24 h after the last dose of IL‐33, and prepared for MELC (Figure S2A). Our subsequent data analysis included various spatial approaches, as laid out in Figure S2B.

### 2.2 Major Immune and Nonimmune Cells Are Annotated from Cyclic IF Data Using the Seurat Workflow

**FIGURE 2:**
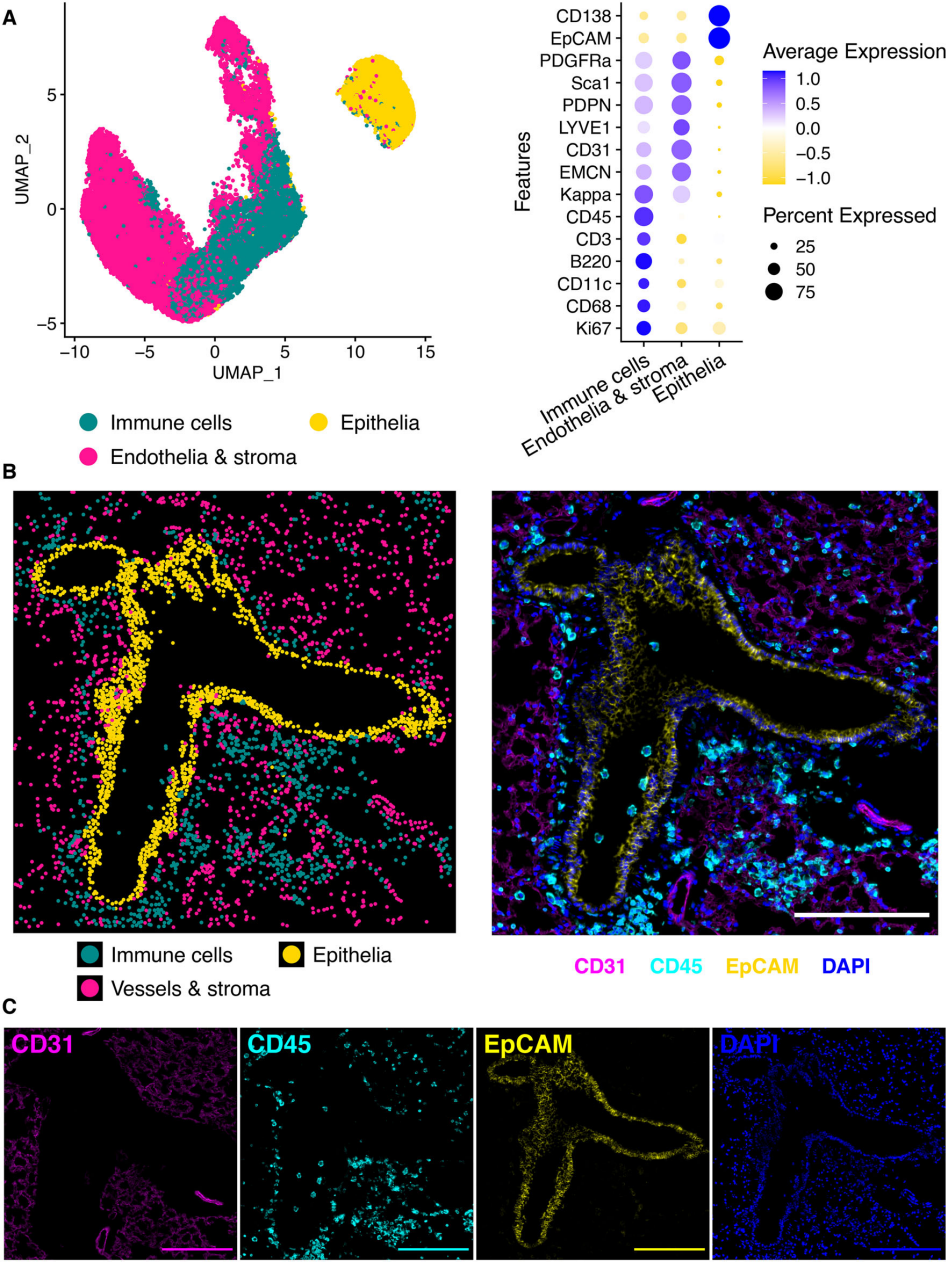
Identification of cell types in AL1 in mouse lungs. (A) UMAP representation of the first level of annotation (AL1) in mouse lung tissue, showing the three clusters annotated as immune cells, endothelia, stroma, and epithelia based on the feature profiles shown in the dot plot beside. The size of the dots in the dot plot correlates with the percentage of cells expressing the respective marker, while the color represents the average expression level of the respective marker by the cluster. (B) Centroids of annotated clusters of AL1 mapped in X and Y space (left) next to IF overlay of the same tissue region (right). Annotations (right) are colored by cell types with immune cells (cyan), endothelia and stroma (magenta), and epithelia (yellow). IF overlay (right) of CD45 (cyan), CD31 (magenta), EpCAM (yellow), and DAPI (blue) of the same FOV. (C) Single marker images of the same tissue region shown in (B) depicting CD31 (magenta), CD45 (cyan), EpCAM (yellow), and DAPI (blue). Scale bar in (B) and (C) represents 200 µm.

We acquired MELC data of mouse lung samples 1, 2, and 3 days after IL‐33 application, as well as healthy controls. After the extraction of single‐cell information by segmentation, dimensionality reduction, and cluster analysis was performed, resulting in three clusters for the first level of annotation (AL1) based on the respective feature profiles (Figure 2A). Thereby, immune cells showed high levels of the pan‐leukocyte marker CD45, as well as various markers for T cells, B cells, plasma cells, and myeloid cells, such as CD3, B220, kappa light chain, and CD68, respectively (Figure 2A). The cluster annotated as endothelia and stroma was high in endothelial‐ and fibroblast‐associated markers EMCN, CD31, LYVE1, podoplanin (PDPN), Sca1, and PDGFRa, while the cluster annotated as epithelia was marked by high levels of EpCAM and CD138 (Figure 2A). Visualization of centroids of annotated immune cells (dark cyan), stromal cells (dark magenta), and epithelia (gold) in *x* and *y* space correlated with the MELC IF overlay of CD45 (cyan), CD31 (magenta), and EpCAM (yellow) of the same tissue area (Figure 2B,C).

**FIGURE 3:**
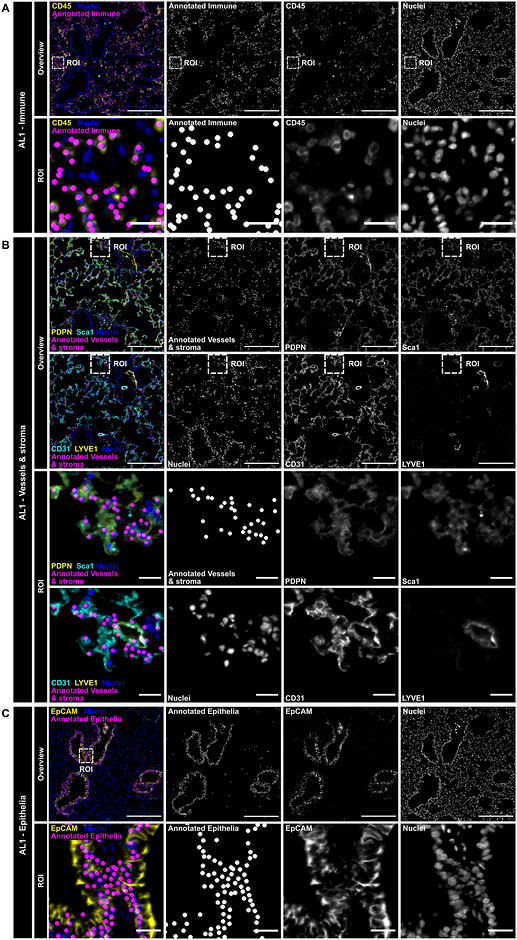
Visual validation of AL1 cell types. (A) Visual validation of annotated immune cells of AL1. Centroids of cells from the cluster annotated as immune (magenta) are overlaid in xy with CD45 (yellow) and nuclei stain (blue) of a representative acquired FOV and a selected ROI of the mouse lung. (B) Visual validation of annotated vessels and stroma of AL1. Centroids of cells from the cluster annotated as vessels and stroma (magenta) are overlayed in xy with PDPN (upper panel; yellow), Sca1 (upper panel; cyan), or LYVE1 (lower panel; yellow), CD31 (lower panel; cyan) together with nuclei stain (blue) of a representative acquired FOV and a selected ROI of mouse lung. (C) Visual validation of annotated epithelia of AL1. Centroids of cells from the cluster annotated as epithelia (magenta) are overlayed in xy with EpCAM (yellow) and nuclei stain (blue) of a representative acquired FOV and a selected ROI of the mouse lung. (A–C) Box marks chosen zoomed‐in ROI. ROI: region of interest. Nuclei image refers to Sytox Green or DAPI staining. Scale bar represents 200 µm in overview images and 20 μ in ROIs.

As correct annotation represents the basis for subsequent analysis and its results, we performed an in‐depth visual validation for all three annotated cellular compartments. The centroids of the annotated cell types were superimposed directly on IF overlays and various markers to confirm correct annotation of AL1 cell types (Figure 2A–C). Centroids of the annotated immune cluster localized on top of nuclear signals, and the majority coexpressed CD45^+^ (Figure 3A). Thereby, the stained cellular membranes showed different shapes and sizes matching the known heterogeneity of immune cell populations, for example, small and round lymphocytes and bigger myeloid cells with dendrite‐like morphologies (Figure 2A). The centroids of the cells annotated as vessels and stroma showed co‐expression of different endothelial and stromal markers (Figure 3B). This included the blood endothelial marker CD31 and the lymphatic endothelial marker LYVE1, as well as PDPN and Sca1, known as markers of stromal cells and fibroblasts (Figure 3B). Furthermore, nuclei as well as the staining pattern of the endothelial and stromal markers partly showed elongated morphologies (Figure 3B). Centroids of the cells from the epithelial cluster localized on areas showing EpCAM staining, mainly restricted to round structures representing the luminal epithelial layer of bronchioles (Figure 3C). As this type of visual validation represents a powerful additional verification tool, it was applied in all subsequent annotation steps of this work.

### 2.3 Three ILC Subtypes Are Resolved, and ILC2s Represent the Predominant Subtype in Mouse Lung Tissue

**FIGURE 4:**
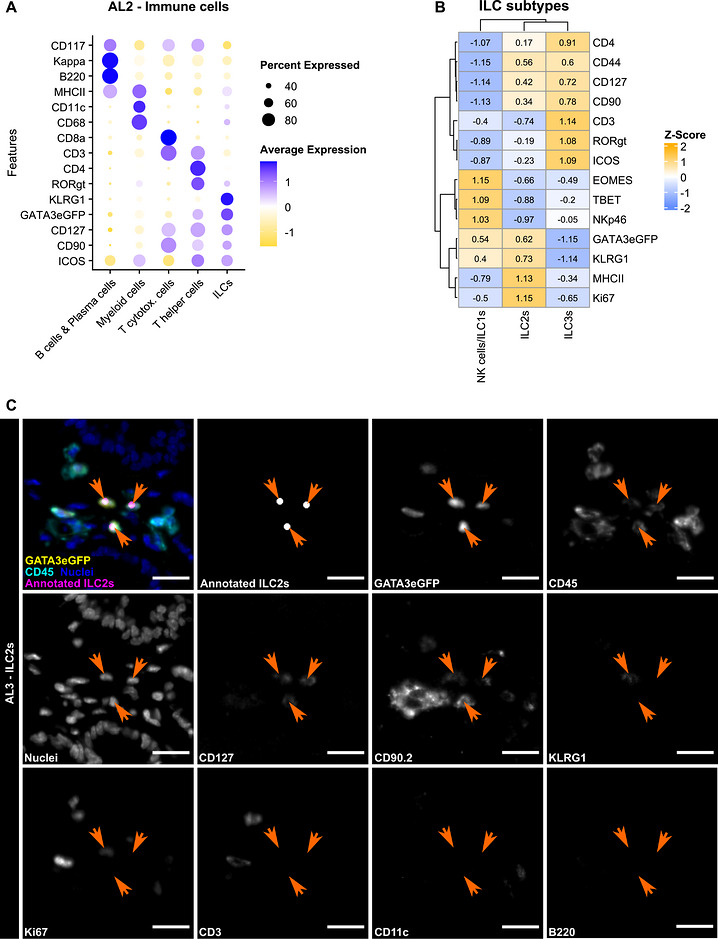
Clustering and annotation of immune cells reveals ILC subtypes. (A) Dot plot showing the marker profiles of the annotated immune cell types in murine lung data. The size of the dots in the dot plot correlates with the percentage of cells expressing the respective marker, while the color represents the average expression level of the respective marker by the cluster. (B) Phenotypic profiling and lineage validation of ILC subtypes. Heatmap representing the mean expression of lineage‐defining and functional markers across identified ILC populations in murine lung tissue. Data are presented as row‐normalized Z‐scores, where orange indicates high relative expression, and blue indicates low relative expression. Numbers within the cells denote the calculated z‐score. Hierarchical clustering (Ward.D2) was applied to both cell types and markers to illustrate phenotypic similarities. (C) Visual validation of ILC2s using IF overlays and single marker stainings of ILC inclusion and exclusion markers. Centroids of annotated ILC2s (magenta) are visualized as dots in xy‐space, each dot representing one ILC2, and overlayed with GATA3eGFP (yellow), CD45 (cyan), and nuclei stain (blue) in the upper left image. Single marker images of GATA3eGFP, CD45, nuclei, CD127, CD90.2, KLRG1, Ki67, CD3, CD11c, and B220 of the same tissue region are depicted in greyscale. Arrows tips (Orange) highlight ILC2s in all depicted images. Scale bar represents 20 µm.

To increase the granularity of our analysis to identify ILC subtypes, we selected cell type‐specific markers and analyzed the two clusters, namely immune, vessels, and stroma, separately. For the immune cells, this approach enabled us to separate five different clusters, which were annotated based on their feature profile (Figure 4A; Figures S3–S5). A CD3^−^ CD127^+^ CD90.2^+^ cluster was annotated as ILCs and showed high levels of GATA3eGFP and KLRG1 (Figure 4A). Other immune cell types of AL2 included B cells and plasma cells (Figure S3), myeloid cells (Figure S4), T cytotoxic cells (Figure S5A), and T helper cells (Figure S5B). We were able to identify three ILC subtypes and annotated the clusters based on their feature profiles. Within the ILC subtypes, NK cells/ILC1s showed high z‐scores of EOMES, TBET, and NKp46 compared with ILC2s and ILC3s. At the same time, ILC2s had the highest z‐scores of GATA3eGFP, KLRG1, MHCII, and Ki67 (Figure 4B). Both ILC2s and ILC3s showed higher expressions of CD127 and CD90.2 compared with NK cells/ILC1s (Figure 4B). ILC3s were annotated based on high RORγt expression. Of note, the annotated ILC3 cluster showed CD3 contamination, suggesting an abundance of Th17 cells within this cluster (Figure 4B). Visual validation of the annotated ILC subtypes with respective ILC‐related markers using IF overlays was performed and confirmed correct annotation (Figure 4C; Figure S6). Besides their nuclear GATA3eGFP signal, annotated ILC2s were positive for CD45, CD127, and CD90.2, and co‐expressed markers such as KLRG1 and Ki67, while being negative for ILC lineage exclusion markers CD3, B220, and CD11c (Figure 4C).

We also performed re‐clustering of the stromal compartment, which resulted in three subtypes: EMCN^+^ CD31^+^ blood vessels, LYVE1^+^ CD90.2^+^ lymphatics, and a LYVE1^+^ CD31^+^ vessel cluster (Figure S7). In summary, we were able to identify 11 different immune and nonimmune cell types in mouse lung MELC data, including the ILC subtypes NK cells/ILC1s, ILC2s, and ILC3s (Figure S8).

**FIGURE 5:**
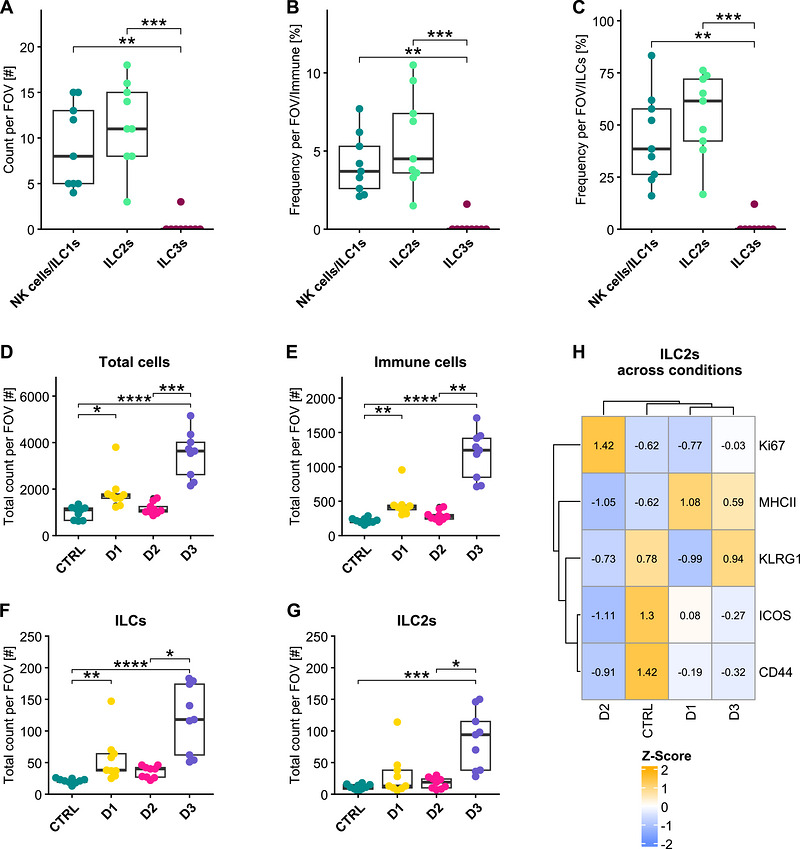
Quantification of total counts and frequencies of cells in mouse lung FOVs across conditions. (A) Box plot depicting the total count of ILC subtypes per FOV at homeostatic conditions. (B) Box plot depicting the frequency of ILC subtypes within the immune compartment per FOV under homeostatic conditions. (C) Box plot depicting the frequency of ILC subtypes within the ILC compartment per FOV under homeostatic conditions. (D–G) Box plots showing the total count of all annotated cells, immune cells, ILCs, and ILC2s per FOV across analyzed conditions, respectively. (H) Phenotypic adaptation of ILC2s across inflammatory conditions. Heatmap displaying the relative expression levels of activation and proliferation markers in ILC2s across control (CTRL) and IL‐33 treatment time points (D1–D3). Data represent pseudo‐bulk average expression, z‐score normalized per marker (row). Hierarchical clustering of markers and conditions was performed using the Ward.D2 method. The color scale indicates the z‐score, ranging from low (blue) to high (orange) relative expression. Individual values within cells denote the calculated z‐score. (A–G) FOV = analyzed fields of view; *n* = 9/analyzed condition; each dot represents one analyzed FOV. For statistical analysis, the Kruskal–Wallis test was used to check for significance between tested groups and effect size, and Dunn's test was used as a post hoc test for pairwise comparison. Asterisks mark significance levels.

Baseline quantification confirmed both ILC2s and NK cells/ILC1s as the predominant ILC subtypes in homeostatic mouse lungs (Figure 5A–C). Consistent with established dissociative analyses, systemic IL‐33 challenge triggered a robust inflammatory influx that peaked at day 3, significantly increasing the total counts of total cells, immune cells, ILCs, and all ILC subtypes per FOVs (Figure 5D–G; Figure S9A,B). Our analysis revealed ILC2s as the predominant ILC subpopulation at IL‐33 day 3 (Figure S9C,D). Looking at their marker expression across conditions, ILC2s at homeostatic conditions showed the highest z‐scores for ICOS and CD44, and also KLRG1 was higher than at IL‐33 days 1 and 2, while MHCII, Ki67, and KLRG1 z‐scores were highest after initiation of the inflammation with IL‐33 (Figure 5H). These findings validate our multiplex workflow's ability to capture shifts in cellular phenotypes, providing the foundation for investigating the spatial organization of these populations.

### 2.4 ILC2s Localize in Mixed Myeloid‐Lymphatic Endothelial Cell Niches

**FIGURE 6:**
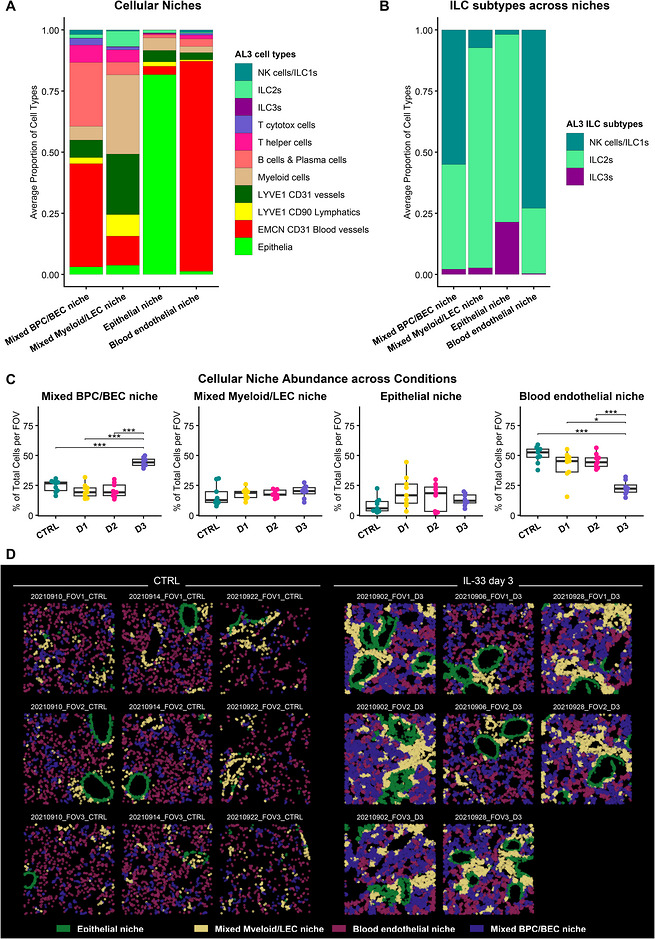
Spatial distribution of tissue niches in mouse lungs across conditions. (A) Stacked bar plot depicting the identified niches and their composition of AL3 cell types. (B) Stacked bar plot depicting the identified niches and their composition of ILC subtypes. (C) Niche abundance across conditions. Each dot represents one acquired FOV. For statistical analysis, the Kruskal–Wallis test was used to check for significance between tested groups and effect size, and Dunn's test was used as a post hoc test for pairwise comparison. Asterisk marks the significance level. *N* of analyzed FOVs: 9 for CTRL, D1, and D2; 8 for D3. FOV: fields of view. (D) Spatial distribution of tissue niches in acquired CTRL and D3 FOVs. Each dot represents the centroid of a cell color‐coded by corresponding tissue niche: epithelial niche (green), mixed myeloid/LEC niche (yellow), blood endothelial niche (magenta), and mixed BPC/BEC niche.

Next, we investigated tissue niches, their cellular composition, and alterations upon IL‐33 injection on a tissue level. For this, we performed niche analysis within 20 µm neighborhoods (Figure 2B,I) of the 11 identified cell types. This resulted in four niches, which were named by their predominant cellular profiles (Figure 6A). While epithelial cells (81.7%) and blood endothelial cells (85.9%) were clearly dominating their respective niches, the other two resulting niches showed a more mixed cellular composition of immune and nonimmune cells (Figure S10A). One of them also comprised a high proportion of blood endothelial cells (42.2%) together with abundant B cells and plasma cells (26.1%), hence named mixed B cell plasma cell/blood endothelial cell (BPC/BEC) niche (Figure 6A). The other mixed niche showed a more diverse composition, with myeloid cells being the most abundant population (32.4%), and LYVE1‐expressing cell types LYVE1 CD31 vessels (24.7% of total cells) and LYVE1 CD90.2 lymphatics (8.8% of total cells), and is therefore termed Myeloid/Lymphatic endothelial cell (LEC) niche (Figure 6A).

Regarding ILCs, the mixed myeloid/LEC niche contained the highest proportion of ILC2s (6.3% of total cells), their numbers being significantly higher compared with all other niches (Figure 6A,B; Figure S10B), indicating a preferred spatial neighborhood of ILC2s. Within the mixed myeloid/LEC niches, ILC2s were the predominant ILC‐subtype at 90.0% (Figure 6A,B). The lowest proportion of ILC2s was present in the blood endothelial niche (0.4% of total cells). However, they still represented around one fourth of the ILC subtypes (26.7% of total ILCs; Figure 6B). On the other hand, NK cells/ILC1s were mainly present in the blood endothelial niche (1.0% of total cells) and the mixed BPC/BEC niche (1.9% of total cells), while being almost absent in the epithelial niche (0.03% of total cells) and the mixed myeloid/LEC niche (0.5% of total cells). Furthermore, NK cells/ILC1s were the predominant ILC subtype in the mixed BPC/BEC niche (55.0% of total ILCs) and the blood endothelial niche (72.9 % of total ILCs). ILC3s, as the rarest of the ILC‐subtypes in our samples, made up less than 0.2% of total cells and all total ILCs in all identified niches, except for the epithelial niche, where they represented 0.3% of the total cells. However, within the ILC compartment, they represented 21.4% and were even more frequent than NK cells/ILC1s (1.9% of total ILCs) in the epithelial niche (Figure 6B).

Comparing the abundance of various niches at the different conditions, a significant expansion of the mixed BPC/BEC niche at IL‐33 day 3 was observed, while at the same time, the blood endothelial niche was significantly reduced at IL‐33 day 3 compared with all other time points (Figure 6C; Figures S10C and S11). This was also obvious in the spatial distribution of the tissue niches in the acquired FOVs (Figure 6D). Analyzing the cellular composition within the endothelial niche showed a significant reduction of blood endothelial cells with increasing IL‐33 doses (Figure S12A). At the same time, a significant increase in B cells and plasma cells at IL‐33 day 3 was observed (Figure 12A), indicating a partial transformation of the endothelial niche toward a BPC/BEC niche in the presence of IL‐33. No significant changes for the abundance of the mixed myeloid/LEC niche, as well as for the epithelial niche, were observed (Figure 6C; Figures 10C and S11). However, quantification of the cell composition of the niches per condition revealed a significant increase in the myeloid proportion within the mixed myeloid/LEC niche at IL‐33 day 1, 2, and 3 compared with the CTRL, as well as a significant reduction of lymphatics at IL‐33 day 3 compared with the CTRL (Figure S12B), indicating niche internal adaptations and changes. Frequencies of ILC2s within the niches did not change significantly (data not shown). The high frequency of ILC2s in the mixed myeloid/LEC niche was also conserved upon IL‐33 injections and significantly differed from the frequency within the blood endothelial niche at all tested conditions (Figure S10D), further supporting a preferred localization of ILC2s in the mixed myeloid/LEC niche.

Taken together, our niche analysis results revealed distinct cellular neighborhoods in mouse lungs. Furthermore, a preferred localization of ILC2s in mixed myeloid/LEC niches was observed, while NK cells/ILC1s were localized within the blood endothelial niches.

**FIGURE 7:**
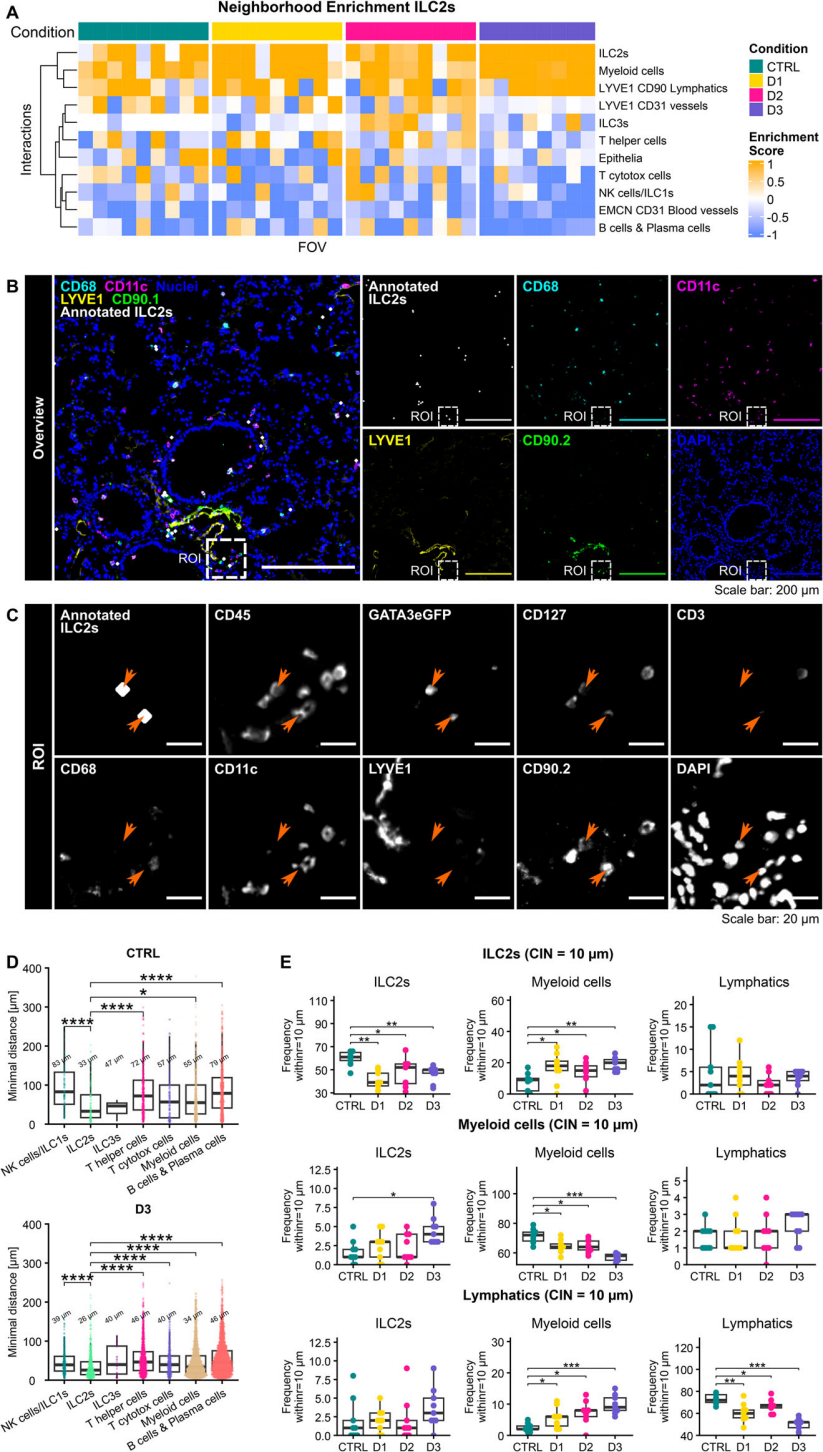
ILC2 localize in niches with myeloid cells and lymphatics. (A) Heatmap showing the results of the neighborhood co‐enrichment analysis of ILC2s with all annotated cell types from AL3 across analyzed conditions as z‐scores. Each column represents one analyzed FOV. (B) Centroids of ILC2s superimposed on IF overlay of the myeloid markers CD68 (cyan) and CD11c (magenta) together with the lymphatic markers LYVE1 (yellow) and CD90.2 (green). White dots mark centroids of identified ILC2s. Each dot represents one identified cell. Box marks ROI. (C) High resolution of zoomed‐in ROI from (B) showing identified ILC2s (Orange arrow heads) superimposed on IF greyscale images of CD45, GATA3eGFP, CD127, CD3, CD68, CD11c, LYVE1, CD90.2, and DAPI. White dots mark centroids of identified ILC2s. Each dot represents one identified cell. (D) Box plots depicting the minimal distances of the identified immune cell types to lymphatics as reference cells under healthy conditions (Top), and D3 (Bottom). Number represents the median minimum distance for the respective cell type in µm. (E) Box plots showing the result of the CIN analysis, showing the frequency of ILC2s, myeloid cells, and lymphatics in a 10 µm radius around ILC2s, myeloid cells, and lymphatics used as reference cells. Each dot represents one cell from nine analyzed FOVs per condition. For statistical analysis, the Kruskal–Wallis test was used to check for significance between tested groups and effect size, and Dunn's test was used as post hoc test for pairwise comparison. Asterisk marks the significance level.

Next, we consolidated our results from the niche analysis and performed extensive spatial analysis focusing on direct cellular interactions of ILC2s and the cell types of the identified mixed myeloid/LEC niche. Spatial coenrichment analysis (Figure S2B II) revealed high coenrichment of ILC2s with other ILC2s, lymphatics, and myeloid cells in tissues at all conditions, but with the highest significance at IL‐33 day 3 (Figure 7A; Figure S12A). This was confirmed by visual inspection, revealing localization of ILC2s close to CD90.2^+^ LYVE1^+^ endothelial structures, as well as myeloid markers that represented lymphatics and myeloid cells, respectively, in IF overlays (Figure 7B). Our examination confirmed direct contact of ILC2s with CD11c and CD68 myeloid cells (Figure 7C). Expanding our panel with additional myeloid markers confirmed the localization of ILC2s in proximity to LYVE1^+^ CD90.2^+^ lymphatics, and the direct contact of ILC2s with CD45^+^ CD11c^+^ CD68^+^ SiglecF^+^ CD44^+^ cells, as indicated by using IF overlays, suggested an activated alveolar macrophage phenotype (Figure S12B). In direct contact with CD11c^+^ myeloid cells within their niche, ILC2s coexpressed markers such as ICOS, MHCII, and KLRG1 (Figure S12C). Of note, ILC2s and lymphatics were both rare cell types and were present with frequencies of 1%–4% within all identified cells, while blood vessels represented one of the most abundant cell types with frequencies of over 40%. The identified ILC2s localized in the tissue surrounding lymphatics, but only rarely inside those vessel structures. Quantitative analyses confirmed 98.08% of ILC2 centroids having a minimum distance to centroids of lymphatic higher than 5 µm under homeostatic conditions (data not shown). The median minimum distance (Figure S2B III) of ILC2s and lymphatics shrank significantly from 33 µm at homeostatic conditions to 26 µm at IL‐33 day 3 (Figure 7D). With this, the minimum distance of ILC2s to lymphatics was the smallest value of all measured immune cells at both conditions (Figure 7D; Figure S13D), supporting the common spatial preference of ILC2s and lymphatics. The cells in neighborhood (CIN) analysis (Figure S2B IV) revealed a significant increase of the myeloid proportion within a 10 µm radius around ILC2s and lymphatics comparing between CTRL and D3 conditions, while the ILC2 proportion increased significantly within the 10 µm radius around myeloid cells (Figure 7E) supporting the spatial connection of the three cell types as well as the inflammatory adaptations of their shared niche.

While the results from the spatial niche and coenrichment analysis revealed a shared niche of ILC2s with myeloid cells and lymphatics, the results for NK cells/ILC1s suggested a different spatial distribution. NK cells/ILC1 were coenriched with EMCN^+^ CD31^+^ endothelial blood vessels and cells from the B cells and plasma cell cluster (Figure S14A,B); however, coenrichment scores were smaller and showed less significance compared with the results of ILC2s (Figure 7A; Figures S13A and S14A,B). Of note, blood vessels were the most abundant cell type identified (over 40%), while NK cells/ILC1s constituted only 0.9%–1.3% of the identified cells. However, the minimal distance of NK cells/ILC1s to blood vessels was only 10 µm under homeostatic conditions, which was significantly less compared with ILC2s, myeloid cells, and T helper cells (Figure S14C). This distance decreased to only 7 µm at IL‐33 day 3, which represented a significant difference compared with all other immune cell types (Figure S14C,D). Also, CIN analysis revealed a significant increase in the proportion of B cells and plasma cells within a 10 µm radius at IL‐33 day 3 around both NK cells/ILC1s and blood vessels (Figure S14E). The proportion of NK cells/ILC1s around blood vessels increased significantly in inflamed tissues, while the proportion of other NK cells/ILC1s dropped significantly in the vicinity of NK cells/ILC1s at IL‐33 day 3 (Figure S14E). On the contrary, CIN analysis of B cells and plasma cells showed decreasing proportions of other B cells and plasma cells while the proportion of blood vessels increased at IL‐33 day 1 (Figure 14E). This supported the changes in niche composition and abundance from our previous niche analysis (Figure 6; Figures S10C, S11, and S12), suggesting tissue adaptations during IL‐33‐mediated inflammation (Figure 6A,D; Figures S10C, S11, and S12). There was no predominant pattern of spatial coenrichment of ILC3s with any other cell type (data not shown).

These data confirm the results from the niche analysis and highlight a predominant spatial distribution pattern of ILC2s in peri‐lymphatic niches in mouse lungs shared with myeloid cells, creating innate immune hubs that become pronounced in IL‐33‐mediated inflammation. Those peri‐lymphatic niches of ILC2s are distinct from the localization of NK cells/ILC1s in peri‐vascular tissue areas in the mouse lung, suggesting different functional spatial neighborhoods of ILC subtypes.

## 3 Discussion

Accounting for less than 1% of the total immune landscape, ILC subtypes are rare tissue‐resident populations. They quickly adapt to changes in their local microenvironment and serve as pivotal orchestrators of immunity and pathology [53, 54, 55]. Dissociative methods have delivered most of our current knowledge of ILC biology; however, it has not been unraveled how such numerically sparse populations can exert such profound and disproportionate influence. This suggests that their potency may be driven by distinct organization into specialized functional niches.

Our spatial analysis confirms that ILCs are not randomly distributed in the tissue, maintaining the broncho‐vascular localization of ILC2s in the lung previously described by Dahlgren et al. [25]. However, our study extends those findings and demonstrates that ILC2s are organized in peri‐lymphatic niches shared with myeloid populations. This preferred localization of ILC2s together with myeloid cells in peri‐lymphatic niches was shown and indicated by all performed analyses, including the niche analysis of the 20 µm neighborhood around cells, quantifying the proportions of cells within a 10 µm radius around a reference cell (CIN analysis), and coenrichment analysis of direct neighbors defined by Delaunay triangulation (Figure S2B I–IV). While this niche remains conserved during homeostasis and at early time points of IL‐33‐induced inflammation, there are adaptations within inflammatory niches, such as the expression of activation and proliferation markers, suggesting a refinement of the functional composition in response to IL‐33 injections (Figure 5H; Figure S13C).

Here, we demonstrate that systemic IL‐33 application not only triggers the accumulation of ILC2s but also increases their colocalization with myeloid cells. Myeloid cells in our study expressed high levels of CD68, CD11c, and MHCII and most likely represent both alveolar macrophages and dendritic cells [56, 57]. This confirms observations of Dahlgren et al. that report the localization of ILC2s together with CD11c^+^ MHCII^+^ myeloid cells/DCs in lung, liver, and kidney [25]. Furthermore, in our study, ICOS was detected in the clusters of T helper cells (Figure 4A) as well as in ILC2s and ILC3s, but not in NK cells/ILC1s (Figure 4B). Compared across conditions, ILC2s had the highest z‐score of ICOS under healthy conditions (Figure 5H). ICOS regulates ILC2 homeostasis independently of T cells and B cells and is required for the proliferation and accumulation of mature ILC2s in the lung and intestine [58, 59]. It has been shown that ICOS^+^ ILC2s are crucial for tissue protection after various nasal challenges [58, 60]. A shared niche of ILC2s and myeloid cells, along with increased ICOSL levels in alveolar macrophages, has been described by Hrusch et al. [60] at early time points of bleomycin‐induced lung inflammation, and Kamachi et al. [61] hypothesized in their study that decreasing levels of ICOS indicate the interaction of ILC2s and DCs via ICOS‐ICOSL in the lungs [62]. Coenrichment of ILC2s and myeloid cells (Figure 7A; Figure S13A), the observed direct contact (Figure S13B,C), and the lower z‐scores of ICOS of ILC2s at inflammatory time points (Figure 5H) might suggest a similar phenomenon of ICOS^+^ ILC2s and myeloid cells in the lung observed in the analyzed MELC data. Furthermore, flow cytometry studies showed that ILC2s not only possess ICOS but also ICOSL [63], which is important for ILC2 homeostasis [58]. ICOS‐ICOSL binding of ILC2s is a survival signal promoting the proliferation of ILC2s and the secretion of proinflammatory cytokines [63], while interactions of ICOS‐expressing ILC2s with ICOSL^+^ T regs dampen inflammatory hypersensitivity responses [64]. In our coenrichment‐ and niche analysis, ILC2s in peri‐lymphatic niches were not coenriched with T helper cells (Figure 7A). This could suggest an autocrine activation of ILC2s by ICOS‐ICOSL at early time points of IL‐33‐mediated inflammation and could be one more reason for ILC2s to accumulate in distinct regions rather than throughout the tissue. Besides the activation of lung ILC2s, it is well described that i.p.‐injection of IL‐33 on consecutive days triggers a systemic type 2 inflammation with an activation and expansion of ILC2s in various other organs, including the SI, kidney, peritoneum, and liver, and interorgan trafficking of activated ILC2s [44, 49, 51, 52]. Intranasal administration of IL‐33 triggered an activated ILC2 phenotype in the lungs, migrating to the liver [65]. One potential scenario might be that ICOS^+^ ILC2s are primed by ICOSL^+^ ILC2s and ICOSL^+^ myeloid cells in the peri‐lymphatic niche to prepare them for interorgan trafficking. Thereby, the peri‐lymphatic location might enable easy and fast exiting of the tissue via lymphatics immediately after activation. However, this hypothesis has to be addressed in subsequent studies, ideally using a high‐resolution spatio‐temporal approach revealing detailed information about the dynamics within the niche and of potentially migratory ILC2 subtypes in the lung.

MHCII was expressed by ILCs, albeit at lower levels than by myeloid cells and B cells (Figure 4A). ILC2s showed the highest z‐score of MHCII within the ILC compartment (Figure 4B), and the highest z‐scores were observed at days 1 and 3 after IL‐33 injection (Figure 5H). MHCII^+^ ILCs act as nonprofessional antigen‐presenting cells, and both ILC2s and ILC3s have been shown to interact with T cells, for example, in the context of helminth infections in the lungs or for maintaining barrier immunity in the gut [66, 67]. Although we occasionally observed MHCII^+^ ILC2s in close or direct contact with T cells (Figure S12C), our results showed no pattern of coenrichment of ILC2s and T helper cells at early time points of IL‐33‐mediated inflammation (Figure 7A). Taking into account that T helper cells are adaptive immune cells, one explanation could be that T helper cells migrate to the niche at later time points of the inflammation. Coenrichment of ILC2s, lymphatics, and myeloid cells also aligns with studies describing an interplay of those cell types [68, 69, 70, 71, 72]. For instance, lung ILC2s stimulate lymphatics (but not blood endothelial cells) to upregulate CCL21 via leukemia inhibitory factor (LIF)‐LIFR, providing a crucial migratory signal for CCR7^+^ immune cells to exit the tissue via the lymphatics to migrate to lymph nodes [71]. LECs actively secrete chemokines such as CCL21 to attract CCR7^+^ DCs and T cells, and there is a feedback loop of DCs and lymphatics mediated by CCL21 that facilitates transendothelial migration [73]. Our MELC analyses support the concept of strategic positioning of ILC2s together with myeloid cells and lymphatics within functional niches in lungs. Spatial transcriptomics approaches would give information on cytokine production and ligand‐receptor interactions of local cell types, confirming the functionality of the niche and its adaptation during inflammation.

Quantification of the ILC2s in our lung data resulted in a frequency of ILC2s of about 5% within the immune compartment under homeostatic conditions. This was much higher than what we expected based on published studies using flow cytometry. In general, the number of ILC2s highly depends on age, sex, and other environmental conditions [74]. In mouse lungs, it is considered very low; for example, Sadeghalvad et al. [48] report an ILC2 frequency of around 0.15% within the immune cell compartment of homeostatic mouse lungs. One explanation for those differences might be the regions we selected for imaging, where we focused on vessels and epithelial structures after having prescreened large tissue areas for the abundance of ILCs. An increased frequency of ILC2s in those peri‐lymphatic niches, despite their low abundance on the whole organ level, may explain how rare cell types can have an important impact on local microenvironments. It also highlights the importance of spatially resolved studies of cells in the tissue context, as this information gets lost during dissociative procedures. Follow‐up studies exploiting spatial technologies with larger acquisition areas might reflect the differences of ILC2 accumulation in peri‐lymphatic niches versus parenchymal areas in a more global way.

While lung ILC2s reside in peri‐lymphatic niches at early time points of IL‐33‐mediated type 2 inflammation, our results suggested lung NK cells/ILC1s to localize in parenchymal regions coenriched with endothelial blood vessels marked by EMCN and CD31, as well as B cells and plasma cells. Lung NK cells and ILC1s have both immunomodulatory functions and roles in pathology and are potent producers of IFNγ [75]. Cautivo et al. [76] showed a de novo accumulation of ILC2s in parenchymal regions 30 days post IL‐33 injections and helminth infection. ILC2s have been reported to express IFNγR, and are responsive to IFNγ secreted by ILC1s and NK cells, for example, in the context of viral infection [77, 78]. It has been shown that IFNγ is a counter signal to IL‐33 that inhibits ILC2 activation and suppresses type 2 immunity in the context of IL‐33‐induced inflammation in mice [79]. Spatially distinct tissue niches of ILC2s and NK cells/ILC1s might represent a natural safety mechanism to avoid unwanted interactions during homeostasis and early time points of type 2 inflammation; however, regulating ILC2s at later time points of inflammation might help to avoid an overshooting or chronic ILC2 activation. In general, subsequent studies characterizing the ILC2 niche at later time points of inflammation would be desirable to see how the described parenchymal localization of ILC2s would affect the niche composition. It is also not clear whether parenchymal ILC2s originate from other lung ILC2s that change localization or whether they immigrate from other organs. The increase in ILC2 frequency at later time points of the inflammation might be ILC2 coming from other organs, such as the intestine. For example, inflammatory ILC2s from the gut enter the lymphatics, then the blood circulation, and migrate to the lung after IL‐25 stimulation and during helminth infection [39, 80]. Migratory ILC2s in the lung increase at around day 5, suggesting an arrival at later time points than what we studied in our IL‐33 model [81]. Of note, this was mainly studied in helminth infections and by administration of IL‐25, which triggered IL‐25R^+^ inflammatory ILC2s, while administration of IL‐33 leads to an activation of ST2^+^ natural ILC2s. This could be studied subsequently by including early and later time points of inflammation using time‐resolved approaches, such as intravital microscopy [82, 83].

While we were mainly focused on the spatial niche characterization of ILC2s in this study, we also observed some global adaptations of the identified niches, including significant expansion of the BPC/BEC niche and a decrease of the blood endothelial niche (Figure 6C). These significant changes appeared at day 3 after IL‐33 application. Especially, the endothelial niche showed changes that might point toward remodeling. For example, within the blood endothelial niche, B cells and plasma cells, myeloid cells, and LYVE1 CD31 vessels increased significantly at IL‐33 day 3, while at the same time, blood endothelial cells decreased in proportion (Figure S10C; Figures S11 and S12A) suggesting a transformation of the predominant blood endothelial niche at homeostatic conditions toward a BPC/BEC and mixed myeloid/LEC niche under the influence of systemic IL‐33 injections. Again, follow‐up studies focusing on the additional later time points will be helpful to understand how local niches are influenced by incoming immune cells from the periphery.

Overall, our study applied a workflow optimized to study rare immune cells and their microenvironment in the context of inflammation and reveals subtype‐specific tissue niches of ILCs. The shared niche of ILC2s, myeloid cells, and lymphatics is preserved upon a systemic type 2 inflammation at early time points but undergoes adaptations. Our methodology can be used as a reference and basis for following investigations of rare cells on a tissue‐scale using spatial multiplex approaches with single‐cell resolution. Furthermore, our study unravels local interaction mechanisms of ILCs that shape the outcome of immunity and pathology [26, 84, 85, 86, 87].

## 4 Limitations

From a technical perspective, working with imaging‐based data points presents several challenges. There is the problem of spatial cross‐contamination in imaging‐based data compared with dissociative methods such as single‐cell sequencing or flow cytometry. Fixed gating strategies are often problematic as thresholds and gates of individual cells are influenced by spatial cross‐contamination of directly neighboring cells and the lateral overlap of 2D technologies. Although we identified 11 different cell types in our dataset, we did not manage to resolve the overall cellular complexity of the lung. High heterogeneity within the lung's stromal compartment is known [88, 89]. We did not resolve fibroblasts as we had high dropout rates of fibronectin (FN) due to technical issues with the machine. Also, our study would have benefited from the inclusion of additional stromal markers, such as different collagens. Furthermore, cytokines are challenging to detect in tissue. High‐resolution spatial transcriptomic approaches would complement our data with valuable information on soluble factors within the niches. Tissues are 3D structures calling for 3D approaches, such as light sheet microscopy, that have the potential to identify hidden complex structures and cellular networks [90, 91, 92], especially for rare cell types. For cell segmentation, we used a combination of pixel classification, segmentation, and feature extraction. This approach was highly resource‐intensive. The use of pretrained AI models [93, 94] has the potential to both reduce the time and enhance the quality and accuracy of the segmentation. Unfortunately, those models often cannot deal with more challenging structures, such as densely packed, inflamed tissues or nonround, complex stromal cells. Newer tools give the possibility to fine‐tune existing models on the datasets [95] or enhance image quality [96]. Although this reduces the time of training, several hours to days must still be invested. Manual visual inspection is a strong advantage when dealing with imaging‐based data compared with dissociative approaches and enables a valuable validation step, but is also very resource‐intensive and laborious.

## 5 Material and Methods

### 5.1 Mice

#### 5.1.1 Ethical Statement

The research presented in this manuscript complies with all relevant ethical regulations. All experimental procedures involving animals were carried out after approval of the study protocols by the Landesamt für Gesundheit und Soziales Berlin (LaGeSo), animal license number (G0122/20). Mice used for this study were kept in the animal facilities of the DRFZ under specific pathogen‐free conditions, which included the maintenance of a 12 h light/dark cycle with the ambient temperature set to 22 ± 2°C and air humidity 55 ± 10% rH. Food and autoclaved water were provided ad libitum. The housing of the animals were cages that were individually ventilated (IVCs) and contained an enriched environment. Animal experiments were conducted following the 3 R Principles: replace, reduce, refine. Mice were handled using tunnels to reduce stress and anxiety.

#### 5.1.2 IL‐33 Application

Female 12‐ to 14‐week‐old GATA3‐enhanced green fluorescent protein (eGFP) reporter mice [97] were i.p.‐injected with 300 ng recombinant carrier‐free mouse IL‐33 (Biolegend, San Diego, USA) dissolved in 0.1 to 0.5 mL sterile saline on up to three consecutive days. The mice were inspected and weighed on a daily basis.

#### 5.1.3 Organ Isolation and Tissue Preparation

Mice were sacrificed after none, 1, 2, or 3 doses of IL‐33, 24 h after the last dose. Approximately 1.5 h before killing the mice, intravenous (i.v.) administration of 200 mg/kg bodyweight Pimonidazole (Hypoxyprobe, Burlington, USA) was performed. Mice were anesthetized and perfused with 1 % freshly prepared electron‐microscopy grade paraformaldehyde (PFA) (EMS, Hatfield, Philadelphia, USA) solution. The lungs were isolated, still connected by the trachea, and incubated for 3 h in 1% PFA solution at 4°C. The PFA solution was discarded, and the samples rinsed with cold PBS for 2–5 min on ice. Afterwards, the organs were incubated in 15 % sucrose solution at 4°C. After 6 to 12 h, the sucrose solution was discarded, and the samples were put into 30 % sucrose solution for 6 to 12 h at 4°C. Lung samples were prepared by inflating the lungs through the trachea with 1:2 PBS:Tissue‐Tek O.C.T. Compound (Sakura) solution using a syringe and carefully put into prefilled cryomolds containing O.C.T. medium.

### 5.2 Cyclic IF: Multiepitope Ligand Cartography (MELC)

#### 5.2.1 Tissue Preparation

Fresh frozen tissue was cut 5 µm thick with an NX80 cryotome (ThermoFisher, Waltham, Massachusetts, USA) on 3‐aminopropyltriethoxysilane (APES)‐coated cover slides (24 × 60 mm; Menzel‐Gläser, Braunschweig, Germany). Samples were fixed for 10 min at room temperature using a freshly opened EM‐grade PFA ampulla (methanol‐ and RNAse‐free; Electron Microscopy Sciences) diluted to 2%. After washing three times with PBS, samples were permeabilized with 0.2% Triton X‐100 in PBS for 10 min at room temperature. Subsequently, a blocking step with 10% goat serum and 1% BSA in PBS was performed for at least 20 min. Afterwards, a fluid chamber holding 100 µL of PBS was created using “press‐to‐seal” silicone sheets (Life Technologies, Carlsbad, California, USA; 1.0 mm thickness) with a circular cut‐out (10 mm diameter), which was attached to the coverslip, surrounding the sample.

For every MELC run, a bottle of fresh PBS with 1% BSA and 0.02% Triton X‐100 was used. The sample was placed on the sample holder and fixed with adhesive tape, followed by accurate positioning of the binning lens, the light path, as well as Köhler illumination of the microscope.

#### 5.2.2 Image Acquisition

The data were acquired using a modified Toponome Image Cycler Mm3 (Tic) (Meltec GmbH & Co. KG, Magdeburg, Germany) and a BioDecipher Device 1.0 (BioDecipher GmbH). Details of equipped components are summarized in Table S1.

MELC image acquisition consists of four steps that are repeated in cycles: (1) Antibody incubation for 15 to 55 min and 30 automatic washing steps. (2) Image acquisition of up to 4 channels of 3 previously selected FOVs. (3) Photo‐bleaching for 5 to 30 min of each FOV. (4) Image acquisition of the bleaching image for every selected FOV. During image acquisition, image stacks of 10 in both positive and negative z‐dimension were acquired, sized 2048 × 2048 pixels, where each pixel represented 0.325 µm.

#### 5.2.3 Antibody Panel

All antibodies used for this study were titrated for the optimal dilution in mouse lung samples and are summarized in Table S2. The optimal order of the antibodies was determined by test experiments. Nuclear dyes DAPI and Sytox green were included as the first and last step, respectively, to evaluate intact tissue quality throughout the experiment. Steric hindrance issues that might appear due to this specific labeling order have been ruled out as previously shown [98].

For each experiment, 60 µL of freshly prepared antibody dilution was pipetted into a 96‐well plate.

#### 5.2.4 Image Preprocessing

Image preprocessing of the Tic MELC data was performed as previously described [24]. For the MELC data acquired with the BioDecipher device, the implemented TICobserver software (BioDecipher GmbH) was used. Both approaches comprised image registration, background subtraction, and illumination correction, and achieved comparable results. Image normalization was performed in Fiji ImageJ [99, 100] just as described in previous publications, and included background estimation (rolling ball algorithm), edge removal, as well as image intensity scaling [24, 31, 98]. Each staining was manually examined for artefacts and excluded when major auto‐fluorescent artefacts covered a dominant area of the stained tissue. If a minor artefact was located in a part of the image where there was no tissue or only covered an area of up to 10 cells, the artefact was cut out from the image, and standardization was reapplied.

#### 5.2.5 Pixel Classification Using Ilastik

Pixel classification was done using Ilastik [101]. The integrated random forest algorithm was trained based on the IF overlay depicting the nuclei (DAPI or Sytox) and a Z‐projection of selected membrane markers (EpCAM, CD45, CD44, CD11c, CD4, LYVE1, podoplanin (PDPN), Sca1, CD68, platelet‐derived growth factor receptor‐α (PDGFRa), CD138, fibronectin (FN), sialic acid‐binding Ig‐like lectin F (SiglecF), and Kappa) to classify pixels into nuclei, cytoplasm, and extracellular matrix (ECM). Training was performed separately for lung and SI data. Visual inspection and minor adjustments of each FOV resulted in optimized probability maps for nuclei, cytoplasm, and ECM that were exported.

#### 5.2.6 Segmentation and Feature Extraction

Probability maps created with Ilastik together with 16‐bit greyscale images were used as input for CellProfiler 4.0 [102] for segmentation of nuclei and cells, as well as for feature extraction and data export. For each FOV of a MELC experiment, the probability maps for nuclei, cytoplasm, and ECM created in Ilastik, as well as all single marker images standardized and intensity‐adapted, were used as input in CellProfiler. Before the actual segmentation, image subtraction was performed by subtracting the ECM probability map from the nuclei probability map as well as from the cytoplasm probability map using CellProfiler's ImageMath module. Subsequently, the subtracted nuclei image was used for the segmentation of nuclei as primary objects with the module IdentifyPrimaryObjects with advanced settings.

By applying an adaptive thresholding strategy and a two‐class Otsu as the thresholding method, segmentation of nuclei was achieved. Using the identified primary objects (nuclei) as seed points and similar settings as for the primary objects, secondary objects were identified by running the IdentifySecondaryObjects module. The identified secondary objects represented cells. Tertiary objects, named cytoplasm, were created by the subtraction of nuclei (primary objects) from cells (secondary objects) implemented in the IdentifyingTertiaryObjects. In case the segmentation outcome did not pass manual inspection, an additional step using the module EditObjectsManually was conducted, where under‐segmented, clothed cell clumps and over‐segmented cells were manually refined.

For each identified object, the median fluorescence intensity (MFI) of nuclear markers was measured in the respective primary object (nucleus), while the MFI of all other markers was measured in the respective secondary object (cell). Resulting from the applied CellProfiler pipeline, the measured single cell features of all objects together with the respective spatial information of the X‐ and Y‐coordinates of the nuclei were exported as CSV files.

Complete and detailed CellProfiler pipelines and all data tables generated are publicly available in the Zenodo open access repository https://zenodo.org/ (see “Data Availability”).

### 5.3 Data Analysis

Data analysis was performed using R [103] and RStudio (RStudio: Integrated Development for R. RStudio, PBC, Boston, MA URL http://www.rstudio.com/) version 2023.06.1. Used packages and versions are summarized in Table S3.

As sometimes not all the markers passed the quality check during the image processing steps, only markers that worked in at least 90 % of the acquired MELC datasets were used for subsequent analysis. In the mouse lung, AREG, B220, CCR6, CD117, CD11c, CD127, CD138, CD3, CD31, CD4, CD44, CD45, CD68, CD8a, CD90.2, endomucin (EMCN), epithelial cell adhesion molecule (EpCAM), ICOS, KLRG1, Kappa, LYVE1, MHCII, NKp46, PDGFRα, PDPN, Sca1, EOMES, GATA3, GATA3eGFP, IRF4, antigen Kiel 67 (Ki67), RORγt, and TBET have been used for downstream analysis. CD134, FN, Gr‐1, IL‐10, IL‐25R, NK1.1, programmed cell death protein 1 (PD1), ST2, CD115, CD200, CD24, CXCR6, SiglecF, vasoactive intestinal polypeptide (VIP), forkhead box protein 3 (FoxP3), intercellular adhesion molecule 1 (ICAM‐1), CD122, GZMA, Perforin, vascular cell adhesion molecule 1 (VCAM‐1), inhibitor of DNA binding 2 (Id2), promyelocytic leukemia zinc finger (PLZF), piezo‐type mechanosensitive ion channel component 1 (Piezo1), and IL‐33 were excluded from the lung MELC data analysis as they did not pass the quality criteria. Of Note, markers that were excluded due to not passing the 90% criteria were still available and useful for the creation of overlays and visual validation of analysis results.

Hyperbolic arcsine (arcsinh) transformation using a cofactor of 2 was applied on the extracted feature data (Wolfram Research (1988), ArcSinh, Wolfram Language function, https://reference.wolfram.com/language/ref/ArcSinh.html; updated 2021). The following strategy was applied for filtering out low‐quality cells: (A) first, the maximum feature value (MFV) from all measured features was identified for each cell. (B) The MFV was visualized in a histogram. (C) The first derivative of the MFV was calculated using Equation (1):

(1) $$\Delta y=y\_\left(i-1\right)-y\_i,$$

with i∈{1,…,*N*},*N* is the maximum value of all MFV, where ∆y represents the change in y from y_(i−1) to y_i. (D) The alteration of the sign of the first derivative of the MFV was identified, representing the threshold. (E) The threshold was applied to the MFV to exclude low‐quality signal cells. (F) The MFV was depicted in a histogram to check the correct application of the threshold. A threshold for the whole mouse lung data set was calculated (0.04) and applied. Data imputation was done using the missRanger package (missRanger: Fast Imputation of Missing Values, Michael Mayer, 2023, https://github.com/mayer79/missRanger). Harmony integration was used for batch correction [104]. Several rounds of the standard Seurat workflow were used for dimensionality reduction, clustering, and cell type annotations in order to identify immune and nonimmune cells.

### 5.4 Spatial Analysis

“Cell in neighborhood (CIN) analysis” (Figure S2B I) was performed using the SPIAT package [105] and applying the function average_percentage_of_cells_within_radius for a radius of 10 µm for every cell type.

“Spatial coenrichment analysis” (Figure S2B II) was performed using the Giotto package [106]. For each FOV, we have employed the createSpatialDelaunayNetwork function to build neighborhood graphs with Delaunay Tesselation. Then we employed the cellProximityEnrichment function to perform coenrichment analysis across cell types. We ran 1000 simulations to calculate the expected cell‐to‐cell proximity frequency compared with the observed frequency, which is then used to calculate the enrichment values. We employed the VoltRon [107] package to visualize the localization of cells overlayed to IF overlay plots. We used the combineChannels function to combine multiple channels before using the vrSpatialPlot function to overlay identified cells to the IF images.

The “minimum cell distance” (Figure S2B III) for every cell within a reference cell population to the closest cell of the target cell type was calculated for all annotated cell types using the SPIAT function calculate_minimum_distances_between_cell types.

“Niche analysis” (Figure S2B IV) was conducted for every individual cell in the dataset by defining the local neighborhood of the index cell and all neighboring cells within a 20 µm radius based on their x‐ and y‐coordinates. Each local neighborhood was tabulated by counting the frequency of the 11 annotated cell types. These raw counts were subsequently transformed into composition vectors representing the relative abundance of cell types within each local environment, independent of total cell density. For all acquired FOVs, the resulting composition vectors were aggregated into a master matrix, and k‐means clustering (*k* = 4) was performed to group similar microenvironments.

Remaining visualizations and plots were created mainly using ggplot2 and ggarrange, as well as Adobe Illustrator and Inkscape. The used packages for the analyses are summarized in Table S4.

### 5.5 Statistical Analysis

If not stated differently in the text, for statistical analysis, the Kruskal–Wallis test was performed to detect significant differences between the distributions of groups and the effect size. Dunn's test was used as a post hoc test for pairwise comparison. Adjusted *p*‐values were calculated using the Bonferroni method. Significance levels of adjusted *p*‐values were depicted as asterisks with the following cut points: **** = 1e‐04; *** = 0.001; ** = 0.01; * = 0.05; ns = 1.

## Author Contributions

A.E.H. conceptualized the study. S.K, R.G., R.U., and P.M. performed experiments. S.K. analyzed the data, and S.K., A.R.P., and A.E.H. interpreted the results and wrote the manuscript. S.K., A.R.P., R.A.N., A.M., and A.E.H. discussed the results. R.K. and L.P. provided technical support for the MELC and BioDecipher instruments. All authors reviewed the manuscript.

## Conflicts of Interest

L.P. is affiliated with BioDecipher GmbH, a company that develops systems for multiplexed fluorescence microscopy. The remaining authors declare no competing interests.

## Supporting information




**Supporting File**: eji70223‐sup‐0001‐SuppMat.pdf.

## Data Availability

All data used for this publication and its figures are available in the following GitHub repository and will be available via Zenodo: https://github.com/mikrohscopist/Murine_ILC_niches_lung_SI_IL‐33
