# Supplementary material for: Spatial and Phenotypic Heterogeneity of ILC Subsets in Mouse Lung Under Type 2 Inflammatory Conditions
Source: Eur J Immunol. 2026 Jun 17;56(6):e70223. doi: 10.1002/eji.70223 (PMC13273930; doi:10.1002/eji.70223)
Supplement: Supplementary file 1 — Supporting File: eji70223‐sup‐0001‐SuppMat.pdf. [file EJI-56-e70223-s001.pdf]

Supporting information

Supplementary Table 1: Overview of MELC panel design with included markers for the respective cell types.

MELC panel design

Type	Markers
ILC inclusion	CD45, CD127, CD90.2
ILC exclusion	CD3, B220, CD68, CD11c, Kappa
ILC subtypes	GATA3eGFP, TBET, EOMES, RORgt, KLRG1, NKp46, CCR6, CD117, NK1.1
Functional	ICOS, MHCII, Ki67, AREG
T cell subtypes	CD3, CD8a, CD4
Additional immune subtypes	CD3, B220, CD68, CD11c, Kappa, CD138, IRF4, SiglecF, GR-1, CD44
Endothelia	CD31, EMCN, LYVE1, CD200
Stromal cells	FN, PDPN, Sca1, PDGFRa
Epithelia	EpCAM, CD24

Supplementary Table 2: Overview of technical components of the Toponome Image Cycler Mm3 and the BioDecipher Device 1.0 used for MELC data acquisition.

	**Toponome Image Cycler Mm3 (Tic) (Meltec Gmbh & Co.Kg Magdeburg, Germany)**
Microscope	Leica DMI 6000B (Leica Microsystems GmbH, Wetzlar, Germany)
Lamp	Lamp HXP R120 / 45C –VIS, EL 6000 (Leica Microsystems GmbH)
Objective	HC PL APO 20x/0.80 PH2 (Leica Microsystems GmbH)
Filters	Filter cubes CY5, AHF-Bandpass DAPI, FITC, mOrange (Leica Microsystems GmbH)
Camera	Orca Flash 4.0LT (Hamamatsu Photonics K.K., Hamamatsu City, Japan)
Diluter	TECAN CAVRO XLP3K SR 3P M6 (Tecan GmbH, Crailsheim, Germany)
Syringe	Syringe XLP/XMP 500 µl (Tecan)
Pipes	10619403 2.5*1.5mm 2800mm pipetting tubing (Tecan)
Cooling system	ThermoStat Plus (Eppendorf, Hamburg, Germany)
Robot	XYZW-robot (Cybertron GmbH, Berlin, Germany)

	**BioDecipher Device 1.0 (BioDecipher GmbH, Magdeburg, Germany)**
Microscope	Leica DMi8 (Leica Microsystems GmbH)
Lamp	Leica LED5 (Leica Microsystems GmbH)
Objective	HC PL APO 20x/0.80 PH2 (Leica Microsystems GmbH)
Filters	Filter cubes DAPI, FITC, TXR, Y5 (Leica Microsystems GmbH)
Camera	Flash 4.0 V3 (Hamamatsu Photonics K.K.)
Diluter	TECAN CAVRO XLP3K SR 3P M6 (Tecan GmbH, Crailsheim, Germany)
Syringe	Syringe XLP/XMP 500 µl (Tecan)
Pipes	10619403 2.5*1.5mm 2800mm pipetting tubing (Tecan)
Cooling system	Temperature controlled 96 plate deep well rack (BioDecipher GmbH, Magdeburg, Germany)
Robot	CAVRO OEM, RSP 9000 (Tecan)

Supplementary Table 3: MELC antibody panel overview

Antibody	Company	Clone	Dilution
Areg-PE	Santa Cruz Biotechnology	G-4	1:100
B220-PE	Miltenyi Biotec	REA755	1:200
CCR6-PE	Biolegend	29-2L17	1:100
CD105-PE	eBioscience	MJ7/18	1:300
CD117-PE	Biolegend	2B8	1:25
CD11b-PE	Miltenyi Biotec	REAL113	1:200
CD11c-PE	Miltenyi Biotec	REA754	1:50
CD127-PE	Invitrogen	A7R34	1:100
CD138-PE	Miltenyi	REA104	1:50
CD146-PE	Miltenyi Biotec	LSEC	1:10
CD163-PE	Invitrogen	TNKUPJ	1:300
CD169-PE	Biolegend	3D6.112	1:50
CD19-PE	Miltenyi Biotec	REA749/GD5	1:50
CD200-PE	Biolegend	OX-90	1:50
CD24-PE	Miltenyi Biotec	REA743	1:50
CD25-PE	Invitrogen	PC61.5	1:50
CD3-PE	Invitrogen	145-2C11	1:50
CD31-Alexa488	R and D Systems	polyclonal	1:50
CD4-PE	Biolegend	RM4-5	1:50
CD44-PE	Miltenyi Biotec	REA664	1:50
CD45-PE	Invitrogen	30-F11	1:300
CD49a-PE	Biolegend	HMa1	1:50
CD80-PE	Miltenyi Biotec	REA983	1:50
CD8a-PE	Miltenyi Biotec	REA601	1:10
CD90.2-APC	Biolegend	30-H12	1:200
CXCR6-PE	Biolegend	SA051D1	1:300
DAPI	Roche		1:5000
EMCN-PE	Invitrogen	eBioV.7C7	1:400
EOMES-PE	Invitrogen	Dan11mag	1:50
EpCAM-PE	Biolegend	G8.8	1:400
F480-Alexa647	BIO RAD	A3-1	1:100
FN-PE	Novus Bio	2755-8	1:400
GATA3-PE	BD	L50-823	1:50
GZMA-PE	Invitrogen	GzM-3G8.5	1:100
Gr1-PE	Miltenyi Biotec	REA810	1:20
ICOS-PE	Miltenyi Biotec	REA192	1:50
IL-25R-PE	Biolegend	9B10	1:50
IRF4-PE	Miltenyi Biotec	REA201	1:10
KLRG1-PE	Biolegend	2F1/KLRG1	1:200
Kappa-FITC	DRFZ	187.2	1:400
Ki67-PE	Invitrogen	SolA15	1:200
LYVE1-PE	Invitrogen	ALY7	1:200
Ly6G-PE	Biolegend	1A8	1:300
MHCII-PE	Biolegend	M5/114.15.2	1:100
Mac2-PE	Biolegend	M3138	1:200
NK1.1-PE	Miltenyi Biotec	REA1162	1:50
NKp46-PE	Biolegend	29A1.4	1:25
PD1-PE	Biolegend	29F.1A12	1:50
PDGFRa-PE	Invitrogen	APA5	1:200

Antibody	Company	Clone	Dilution
PDPN-PE	Biolegend	36899	1:400
PRF1-PE	Biolegend	S16009 B	1:50
RORgt-PE	BD	Q31-378	1:50
SMA-FITC	Abcam	1A4	1:50
ST2-PE	proSci	polyclonal	1:200
Sca1-APC	eBioscience	D7	1:200
SiglecF-PE	BD	E50-2440	1:300
Sytox green	Thermo Fisher		1:100
TBET-PE	Biolegend	4B10	1:50
TCRab-PE	Miltenyi Biotec	REA318	1:10
VCAM1-APC	Miltenyi Biotec	REA971	1:50
VIM-Alexa488	Abcam	EPR3776	1:100
anti-GFP-Alexa488	Rockland	polyclonal	1:50
anti-rab-PE	Rockland	polyclonal	1:200

Supplementary Table 4: Overview of R packages and versions used for data analysis.

Data analysis

Package	Version
Seurat	4.3.0.1
SeuratObject	4.1.3
clustree	0.5.0
cowplot	1.1.1
data.table	1.14.8
dplyr	1.1.2
forcats	1.0.0
ggplot2	3.4.4
ggpmisc	0.5.4-1
ggpp	0.5.4
ggpubr	0.6.0
ggraph	2.1.0
ggrepel	0.9.3
glue	1.6.2
gridExtra	2.3
here	1.0.1
lubridate	1.9.2
magick	2.8.0
magrittr	2.0.3
moments	0.14.1
patchwork	1.1.2
plotrix	3.8-2
png	0.1-8
readr	2.1.4
reshape2	1.4.4
rstatix	0.7.2
scales	1.2.1
stringr	1.5.0
viridis	0.6.4
viridisLite	0.4.2

Spatial analysis

Package	Version
Biobase	2.62.0
BiocGenerics	0.48.1
GenomeInfoDb	1.38.1
GenomicRanges	1.54.1
Giotto	4.0.2
GiottoClass	0.1.3
IRanges	2.36.0
MatrixGenerics	1.14.0
S4Vectors	0.40.2
SPIAT	1.4.1
Seurat	5.0.1
SeuratObject	5.0.1
SingleCellExperiment	1.24.0
SpatialExperiment	1.12.0
SummarizedExperiment	1.32.0
VoltRon	1.0.0
clustree	0.5.1
data.table	1.15.0
dplyr	1.1.4
forcats	1.0.0
ggplot2	3.4.4
ggpubr	0.6.0
ggraph	2.1.0
ggrepel	0.9.5
glue	1.6.2
here	1.0.1
lubridate	1.9.3
magrittr	2.0.3
matrixStats	1.2.0
moments	0.14.1
patchwork	1.2.0
plotrix	3.8-4
readr	2.1.5
rlang	1.1.3
rstatix	0.7.2
scales	1.3.0
sp	2.1-3
stringr	1.5.1

Supporting figures

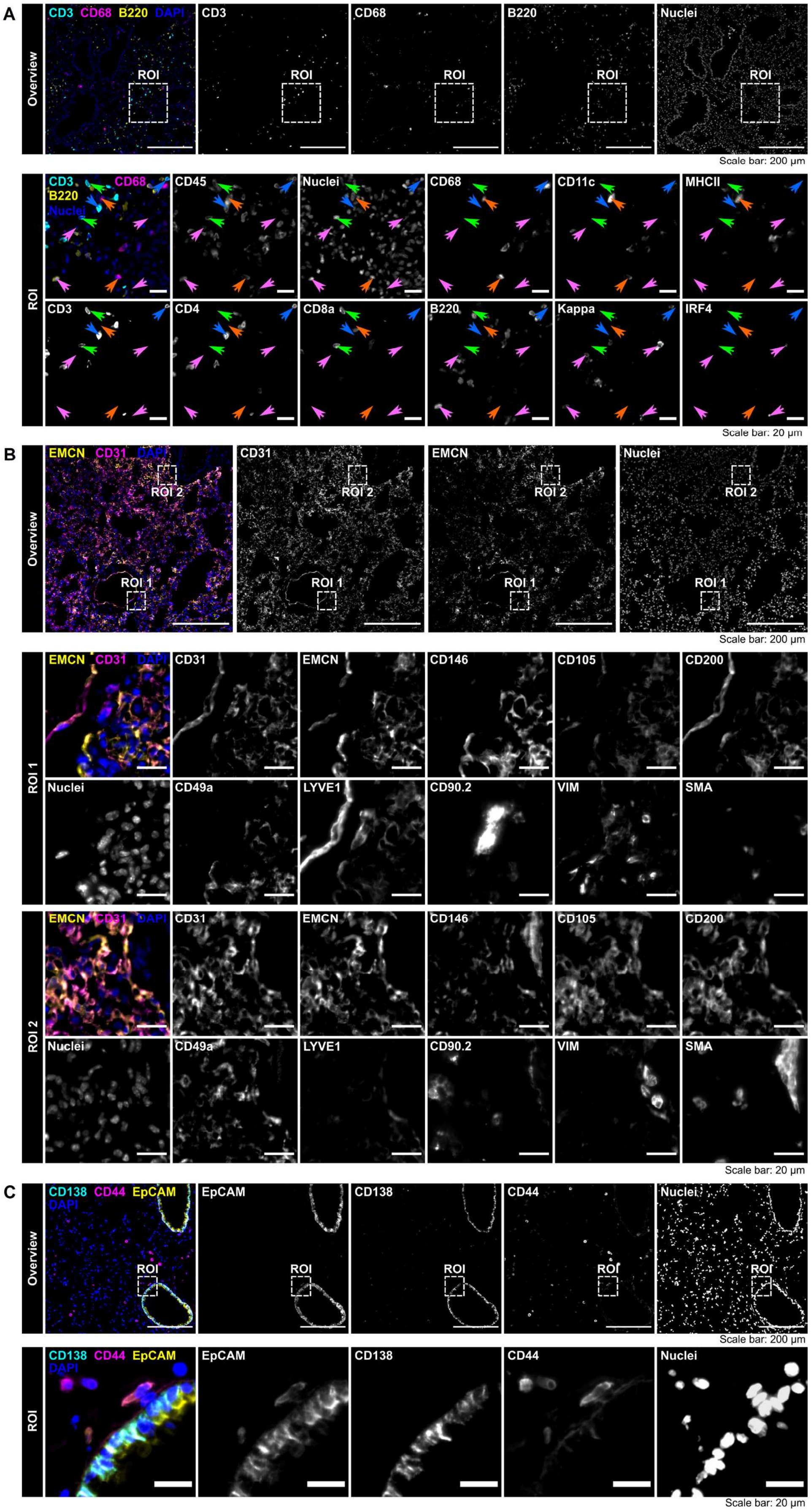
A
Overview
CD3 CD68 B220 DAPI
CD3
CD68
B220
Nuclei
ROI
Scale bar: 200 µm
ROI
CD3 CD68 B220 Nuclei
CD45
Nuclei
CD68
CD11c
MHCII
CD3
CD4
CD8a
B220
Kappa
IRF4
Scale bar: 20 µm
B
Overview
EMCN CD31 DAPI
CD31
EMCN
Nuclei
ROI 1
ROI 2
Scale bar: 200 µm
ROI 1
EMCN CD31 DAPI
CD31
EMCN
CD146
CD105
CD200
Nuclei
CD49a
LYVE1
CD90.2
VIM
SMA
ROI 2
EMCN CD31 DAPI
CD31
EMCN
CD146
CD105
CD200
Nuclei
CD49a
LYVE1
CD90.2
VIM
SMA
Scale bar: 20 µm
C
Overview
CD138 CD44 EpCAM DAPI
EpCAM
CD138
CD44
Nuclei
ROI
Scale bar: 200 µm
ROI
CD138 CD44 EpCAM DAPI
EpCAM
CD138
CD44
Nuclei
Scale bar: 20 µm

Suppl. Figure 1: IF overlays of the designed panel. (A) MELC IF overlays of immune markers. IF overlay of CD3 (Cyan), B220 (Yellow), CD68 (Magenta), and DAPI (Blue) shown for a representative acquired FOV (Upper panel) and a selected ROI (Lower panel) in mouse lung. Besides overlays of the FOV and the ROIs, single marker images of additional immune markers are depicted in greyscale. Arrow heads highlight examples for $CD3^+$ $CD4^+$ T helper cells (Blue), $CD3^+$ $CD8a^+$ T cytotox. cells (Green), $CD68^+$ $CD11c^+$ myeloid cells (Orange) partly expressing MHCII, and cells of the B lineage (Pink) expressing different levels and combinations of B220, Kappa, and IRF4. **(B)** MELC IF overlays of endothelial markers. IF overlay of EMCN (Yellow), CD31 (Magenta), and DAPI (Blue) shown for a representative acquired FOV (Upper panel) and two selected ROIs (Middle and lower panel) in mouse lung. Besides overlays of the FOV and the ROIs, single marker images of additional endothelial markers are depicted in greyscale. **(C)** MELC IF overlays of epithelial markers. IF overlay of EpCAM (Yellow), CD44 (Magenta), CD138 (Cyan), and DAPI (Blue) shown for a representative acquired FOV (Upper panel) and one selected ROI (Lower panel) in mouse lung. (A-C) Scale bar represents 200 µm in overview images and 20 µ in ROIs. Nuclei staining refers to either DAPI or sytox green. EMCN: endomucin; IF: immunofluorescence; MELC: multi epitope ligand cartography; ROI: region of interest; SMA: smooth muscle actin; VIM: vimentin.

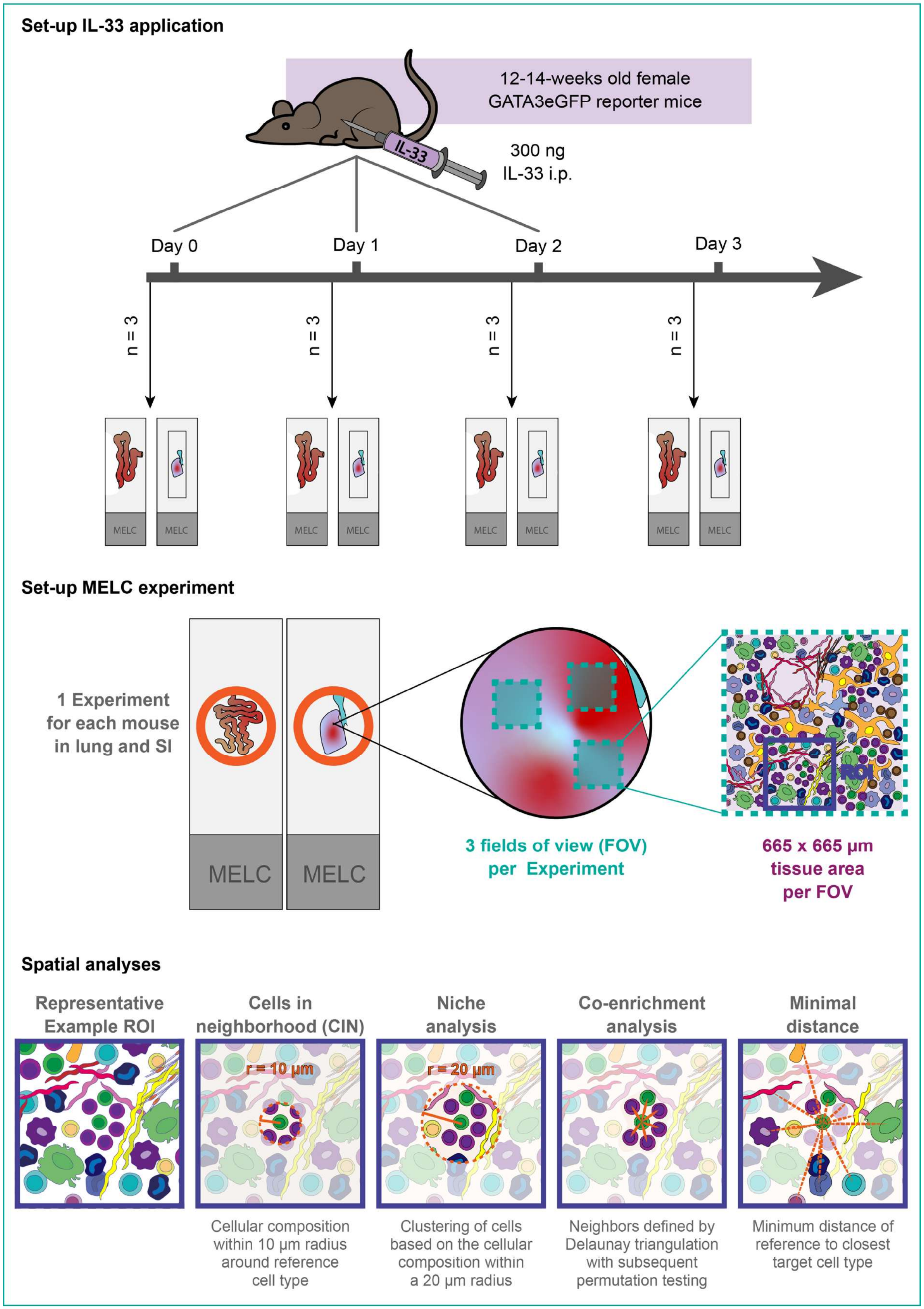

Suppl. Figure 2: Experimental workflow and spatial analyses approaches. (A) Schematic of the experimental set-up of the IL-33 systemic inflammation model and the MELC experiments. In short, 12-14-week-old GATA3eGFP reporter mice were *i.p.* injected with 300 ng IL-33 on up to 3 consecutive days.

24 h after the last dose, organs were harvested and processed for cyclic IF (MELC). In each MELC experiment, three FOVs were acquired, each measuring 665 x 665 µm. Purple box marks ROI shown in (B). **(B)** Analysis of the data comprised different spatial approaches on different scales including niche analysis (I), coenrichment analysis (II), minimum distances (III), and CIN analysis (IV). ROI: region of interest; MELC: multi epitope ligand cartography; CIN: cells in neighborhood.

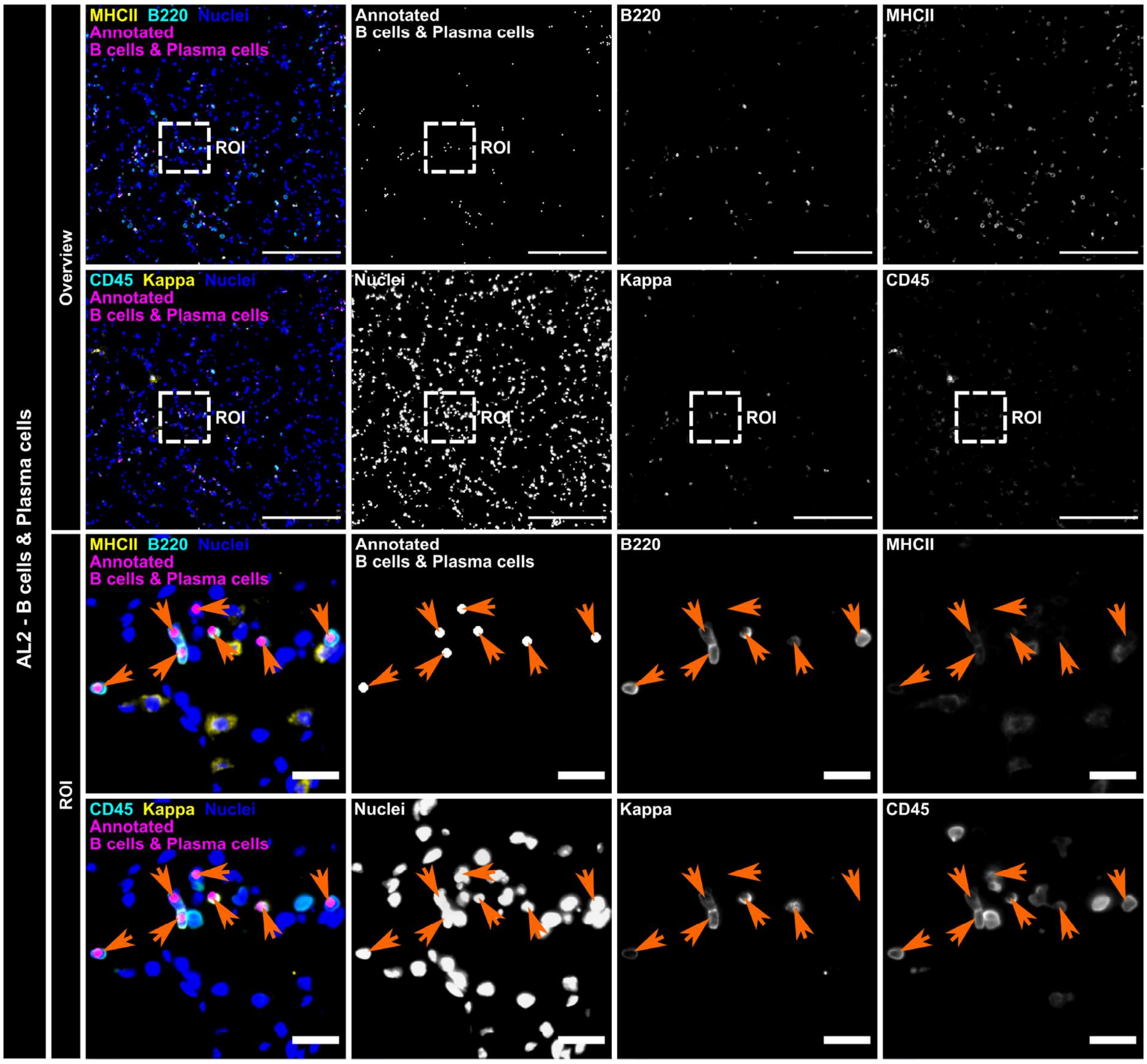

Suppl. Figure 3: Visual validation of annotated B cells & plasma cells using IF overlays and single marker stainings. Centroids of annotated cells are visualized as dots in xy-space (Magenta), each dot representing one cell. IF stainings of MHCII (Upper panel; Yellow), B220 (Upper panel; Cyan), or Kappa (Lower panel; Yellow) and CD45 (Lower panel; Cyan) are shown with nuclei stain (Blue) and cell centroids are superimposed (Magenta). Single marker images of B220, MHCII, kappa, CD45, and nuclei are shown for one representative FOV and a zoomed-in ROI in greyscale. Arrows tips (Orange) highlight identified annotated cells in all depicted ROI images. Scale bar represents 200 µm in FOV and 20 µm in ROI. FOV: field of view; ROI: region of interest.

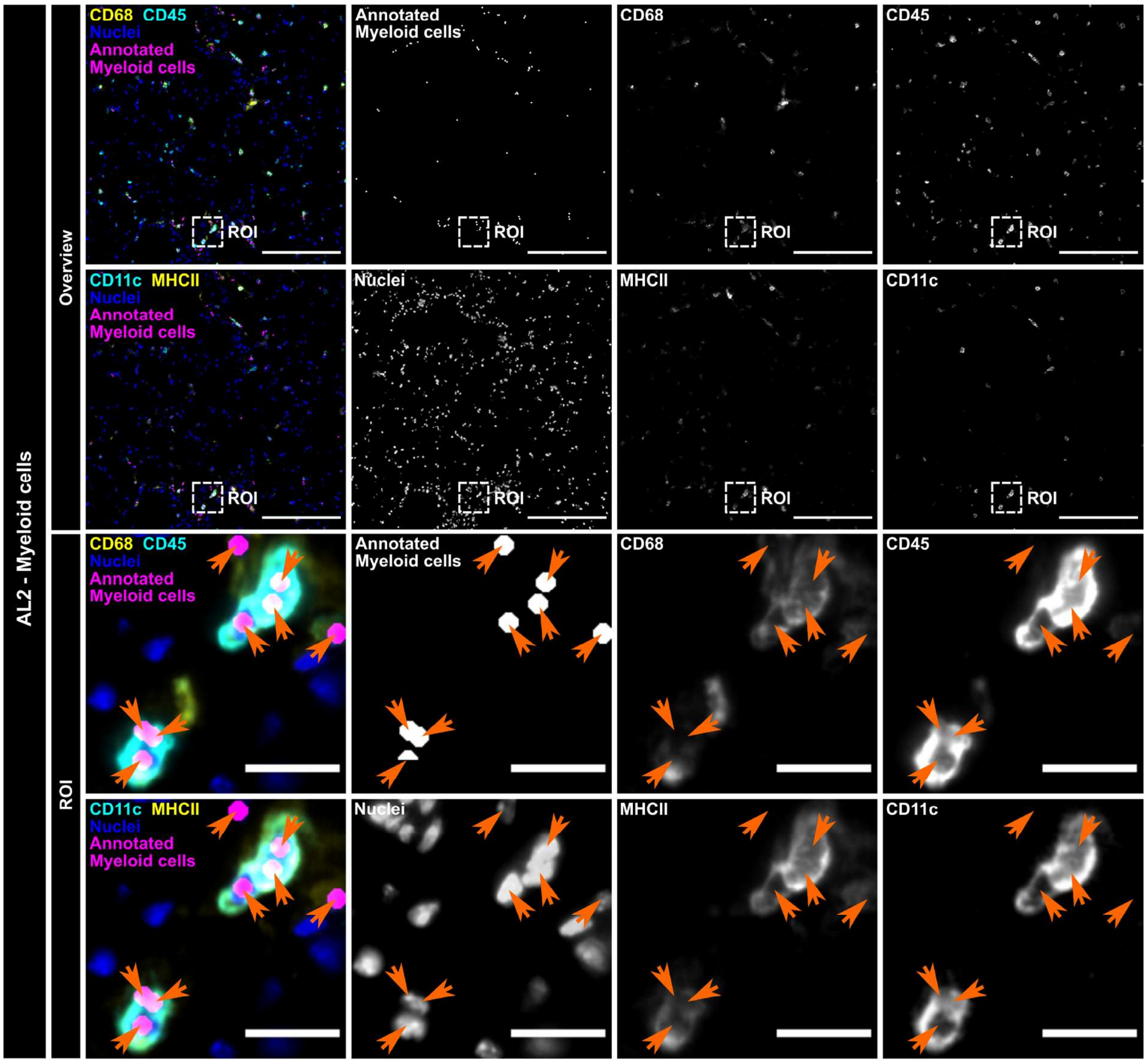

Suppl. Figure 4: Visual validation of annotated myeloid cells using IF overlays and single marker stainings. Centroids of annotated cells are visualized as dots in xy-space (Magenta), each dot representing one cell. IF stainings of CD68 (Upper panel; Yellow), CD45 (Upper panel; Cyan), or MHCII (Lower panel; Yellow) and CD11c (Lower panel; Cyan) are shown with nuclei stain (Blue) and cell centroids are superimposed (Magenta). Single marker images of CD68, MHCII, CD11c, CD45, and nuclei are shown for one representative FOV and a zoomed-in ROI in greyscale. Arrow tips (Orange) highlight identified annotated cells in all depicted ROI images. Scale bar represents 200 µm in FOV and 20 µm in ROI. FOV: field of view; ROI: region of interest.

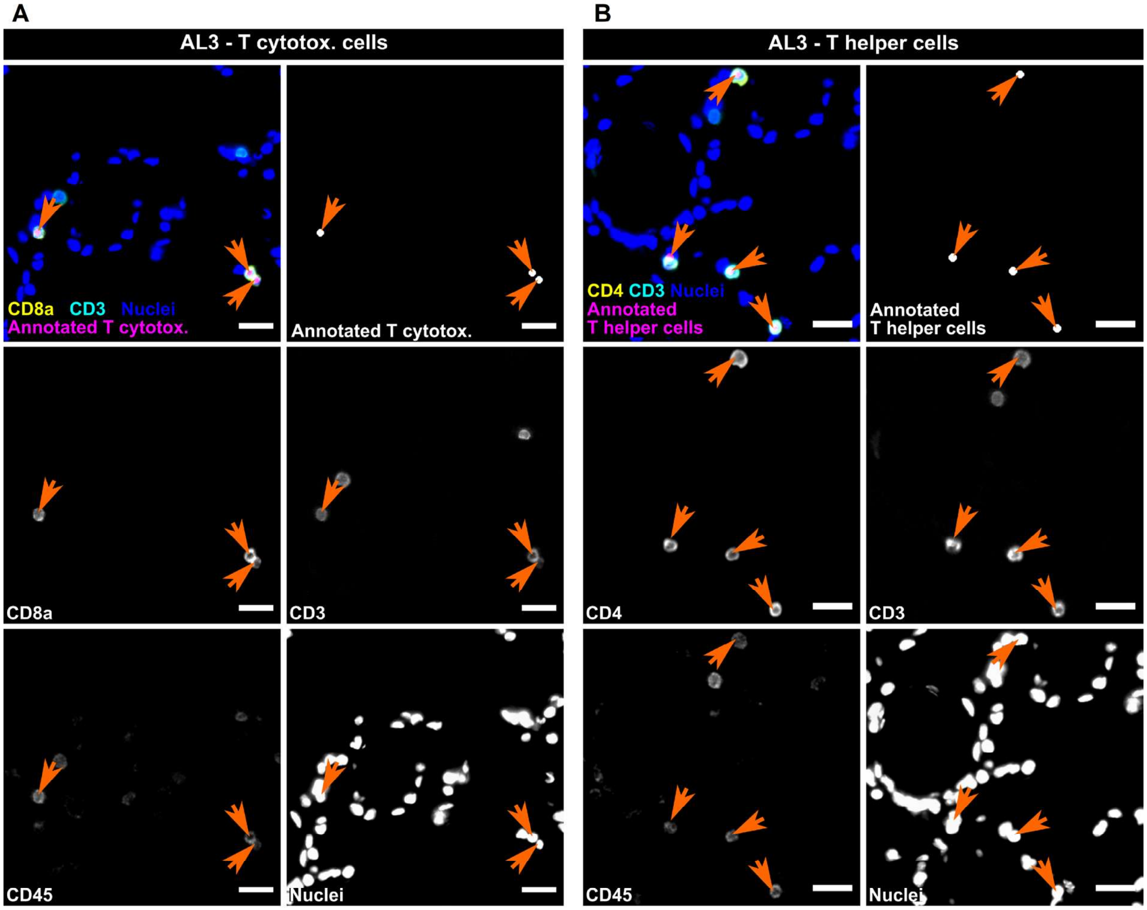

Suppl. Figure 5: Visual validation of annotated T cell subsets using IF overlays and single marker stainings. (A) Visual validation of annotated T cytotoxic cells using IF overlays and single marker stainings. Centroids of annotated cells are visualized as dots in xy-space (Magenta), each dot representing one cell, and overlayed with CD8a (Yellow), CD3 (Cyan), and nuclei stain (Blue) in the upper left image. Single marker images of CD8a, CD3, nuclei, and CD45 of the same tissue region are depicted in greyscale. Arrows tips (Orange) highlight ILC2s in all depicted images. Scale bar represents 20 µm. **(B)** Visual validation of annotated T helper cells using IF overlays and single marker stainings. Centroids of annotated cells are visualized as dots in xy-space (Magenta), each dot representing one cell, and overlayed with CD4 (Yellow), CD3 (Cyan), and nuclei stain (Blue) in the upper left image. Single marker images of CD4, CD3, nuclei, and CD45 of the same tissue region are depicted in greyscale. (A) and (B) Arrows tips (Orange) highlight identified annotated cells in all depicted images. Scale bar represents 20 µm.

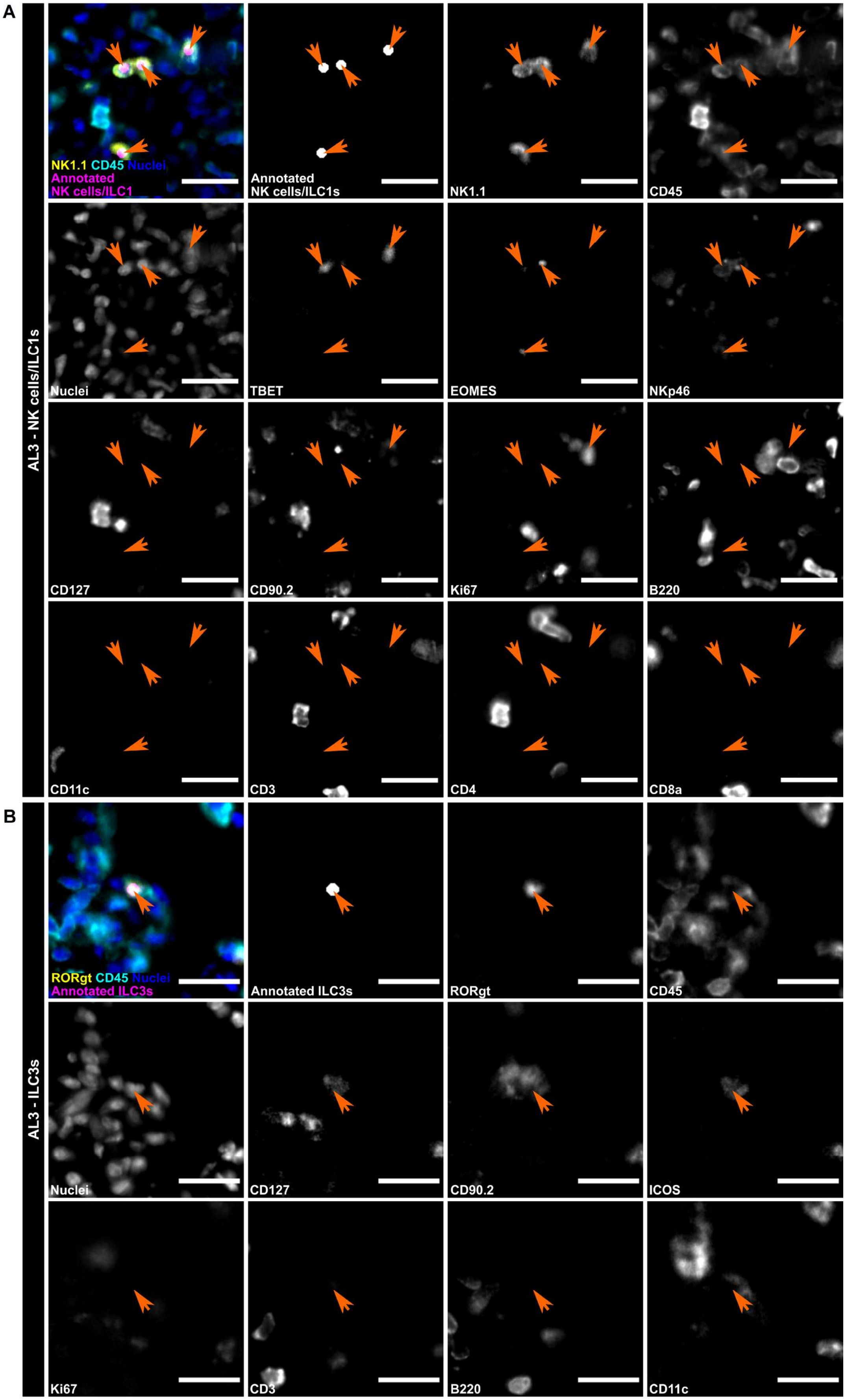
A
AL3 - NK cells/ILC1s
NK1.1 CD45 Nuclei
Annotated
NK cells/ILC1
Annotated
NK cells/ILC1s
NK1.1
CD45
Nuclei
TBET
EOMES
NKp46
CD127
CD90.2
Ki67
B220
CD11c
CD3
CD4
CD8a
B
AL3 - ILC3s
RORgt CD45 Nuclei
Annotated ILC3s
Annotated ILC3s
RORgt
CD45
Nuclei
CD127
CD90.2
ICOS
Ki67
CD3
B220
CD11c

Suppl. Figure 6: Visual validation of NK cells/ILC1s and ILC3s. (A) Visual validation of cells of the NK cells/ILC1 cluster using IF overlays and single marker stainings of ILC inclusion and exclusion markers. Centroids of annotated NK cells/ILC1 (Magenta) are visualized as dots in xy-space, each dot representing one NK cells/ILC1, and overlayed with NK1.1 (Yellow), CD45 (Cyan), and nuclei stain (Blue) in the upper left image. Single marker images of NK1.1, CD45, EOMES, TBET, NKp46, CD127, CD90.2, Ki67, B220, CD11c, CD3, CD4, CD8a, and nuclei stain of the same tissue region are depicted in greyscale. **(B)** Visual validation of ILC3s using IF overlays and single marker stainings of ILC inclusion and exclusion markers. Centroids of annotated ILC3s (Magenta) are visualized as dots in xy-space, each dot representing one ILC3, and overlayed with RORγt (Yellow), CD45 (Cyan), and nuclei stain (Blue) in the upper left image. Single marker images of RORγt, CD45, nuclei, CD127, CD90.2, ICOS, Ki67, CD3, CD11c, B220, and nuclei stain of the same tissue region are depicted in greyscale. (A) and (B) Arrows tips (Orange) highlight annotated cells in all depicted images. Scale bar represents 20 μm.

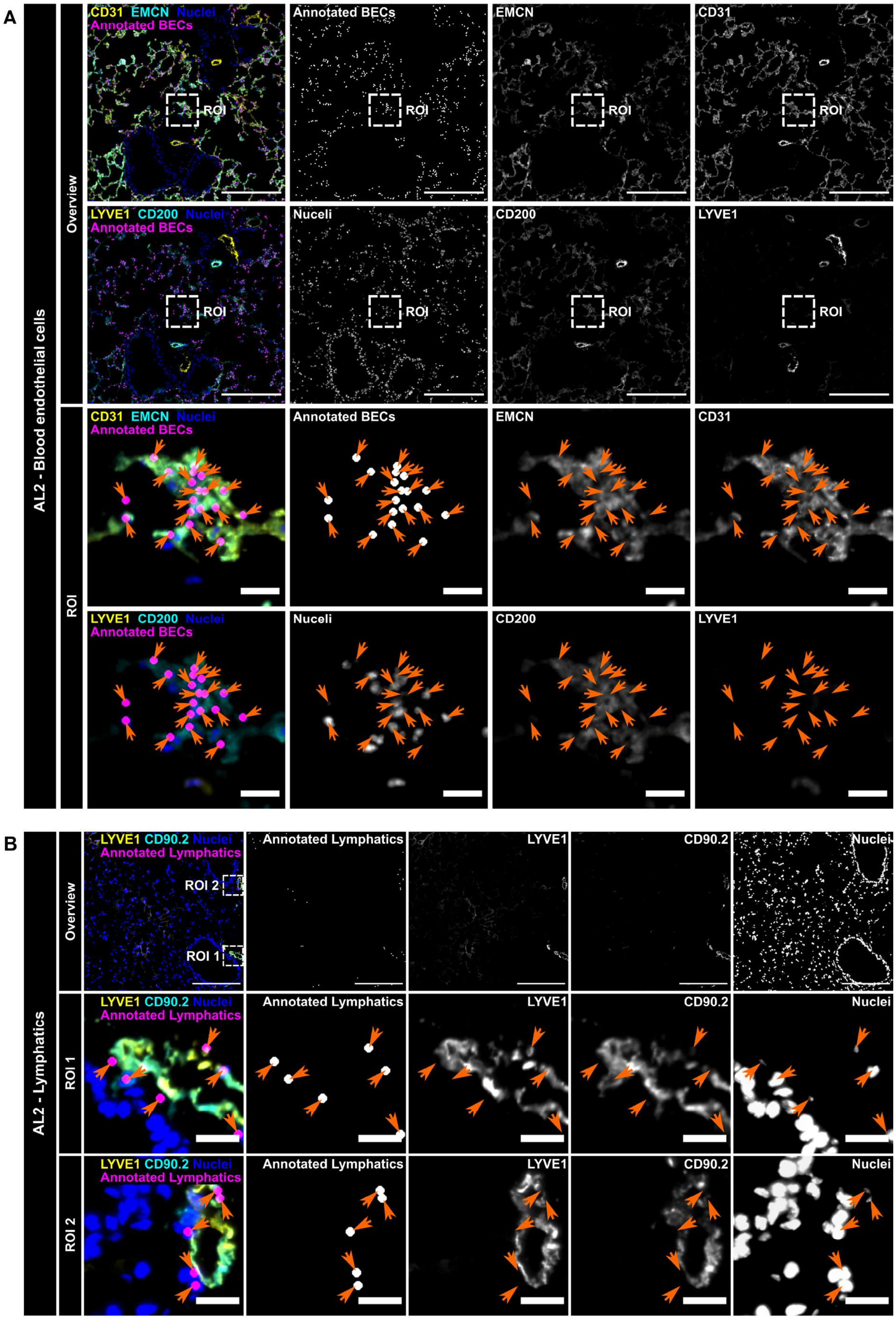

Suppl. Figure 7: Visual validation of endothelial subpopulations. (A) Visual validation of annotated blood endothelial cells using IF overlays and single marker stainings. Centroids of annotated blood

endothelial cells (Magenta) are visualized as dots in xy-space, each dot representing one cell, and overlayed with CD31 (Upper panel; Yellow), EMCN (Upper panel; Cyan), or LYVE1 (Lower panel; yellow), CD200 (Lower panel; Cyan) together with the nuclei stain (Blue). Single marker images of CD31, EMCN, LYVE1, CD200, and nuclei stain of the same tissue region are depicted in greyscale for one representative FOVs and one zoomed-in ROIs marked by white dotted box. **(B)** Visual validation of annotated lymphatics using IF overlays and single marker stainings. Centroids of annotated lymphatics (Magenta) are visualized as dots in xy-space, each dot representing one cell, and overlayed with LYVE1 (yellow), CD90.2 (Cyan) together with the nuclei stain (Blue). Single marker images of LYVE1, CD90.2, and nuclei stain of the same tissue region are depicted in greyscale for one representative FOVs and two zoomed-in ROIs marked by white dotted box. (A) and (B) Arrows tips (Orange) highlight annotated cells in all depicted images. Scale bar represents 200μm in FOVs, and 20 μm in depicted ROIs. EMCN: endomucin; FOV: field of view; ROI: region of interest.

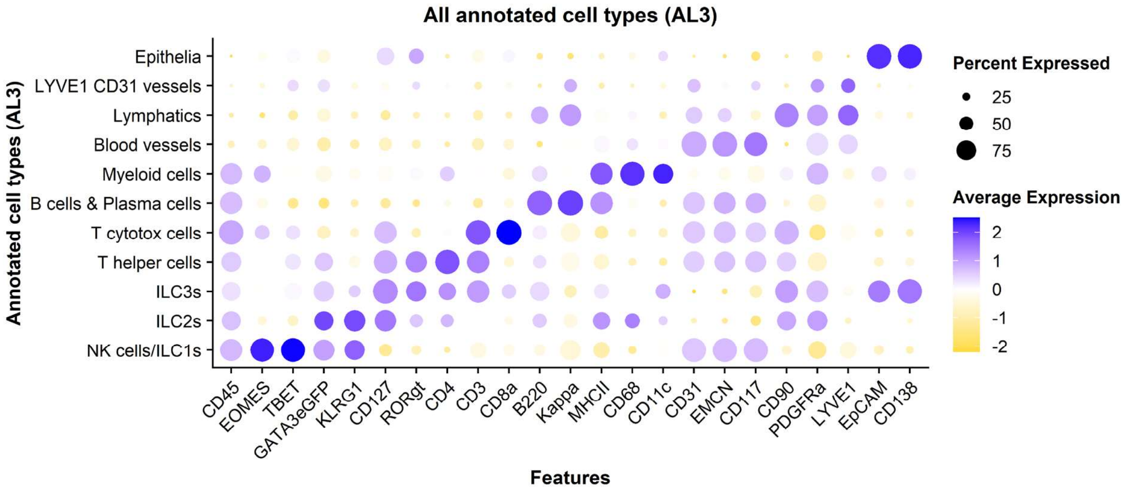

Suppl. Figure 8: Marker profiles of all annotated cell types within AL3. Dot plot showing the marker profiles of all annotated cell types of AL3 in the mouse lung dataset. The size of the dots in the dot plot correlates with the percentage of cells expressing the respective marker, while the color represents the average expression level of the respective marker by the cluster. AL: annotation level.

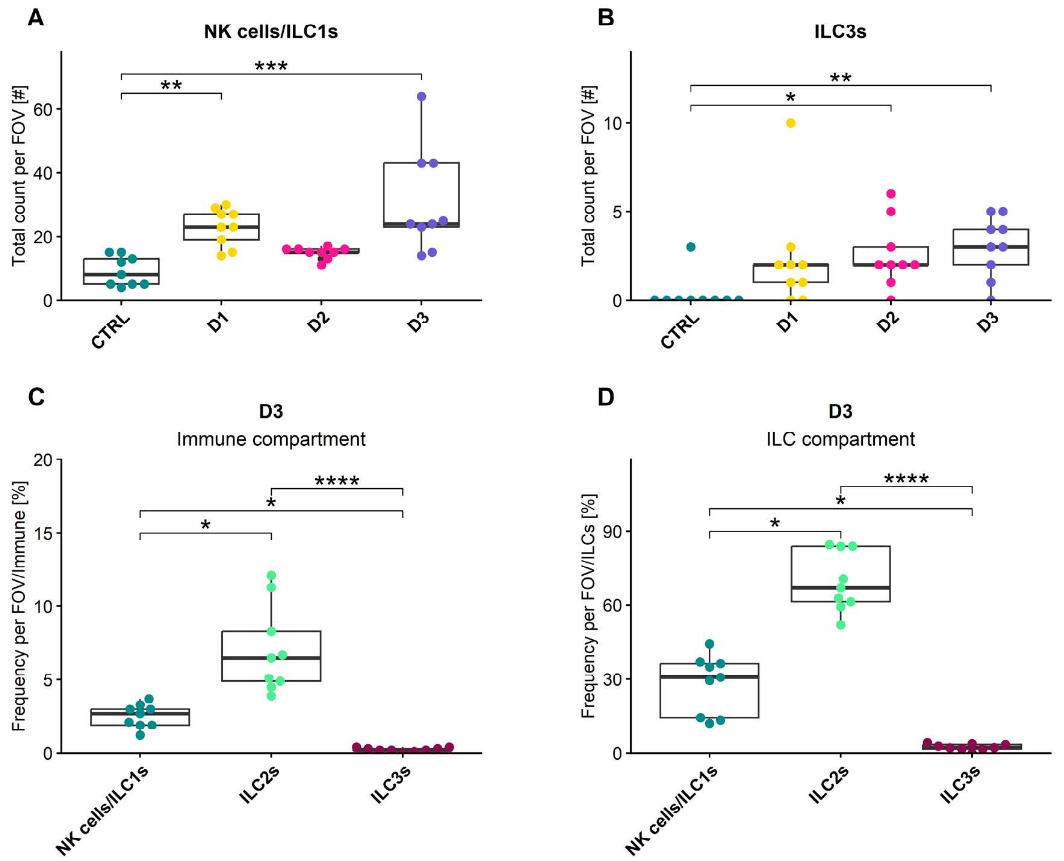

Suppl. Figure 9: Quantification of total counts of NK cells/ILC1s and ILC3s in mouse lung FOVs across conditions. (A) Box plots showing the total count of annotated NK cells/ILC1s per analyzed FOV across conditions. **(B)** Box plots showing the total count of annotated ILC3s per analyzed FOV across conditions. **(C)** Box plot depicting the frequency of ILC subtypes within the immune compartment per FOV for IL-33 day 3 (D3). **(D)** Box plot depicting the frequency of ILC subtypes within the ILC compartment per FOV for IL-33 day 3 (D3). (A-D) FOV = analyzed fields of view; n = 9 for each condition; each dot represents one analyzed FOV. For statistical analysis, Kruskal-Wallis-test was used to check for significance between tested groups and effect size, Dunn's test was used as post-hoc test for pairwise comparison. Asterisks mark significance levels.

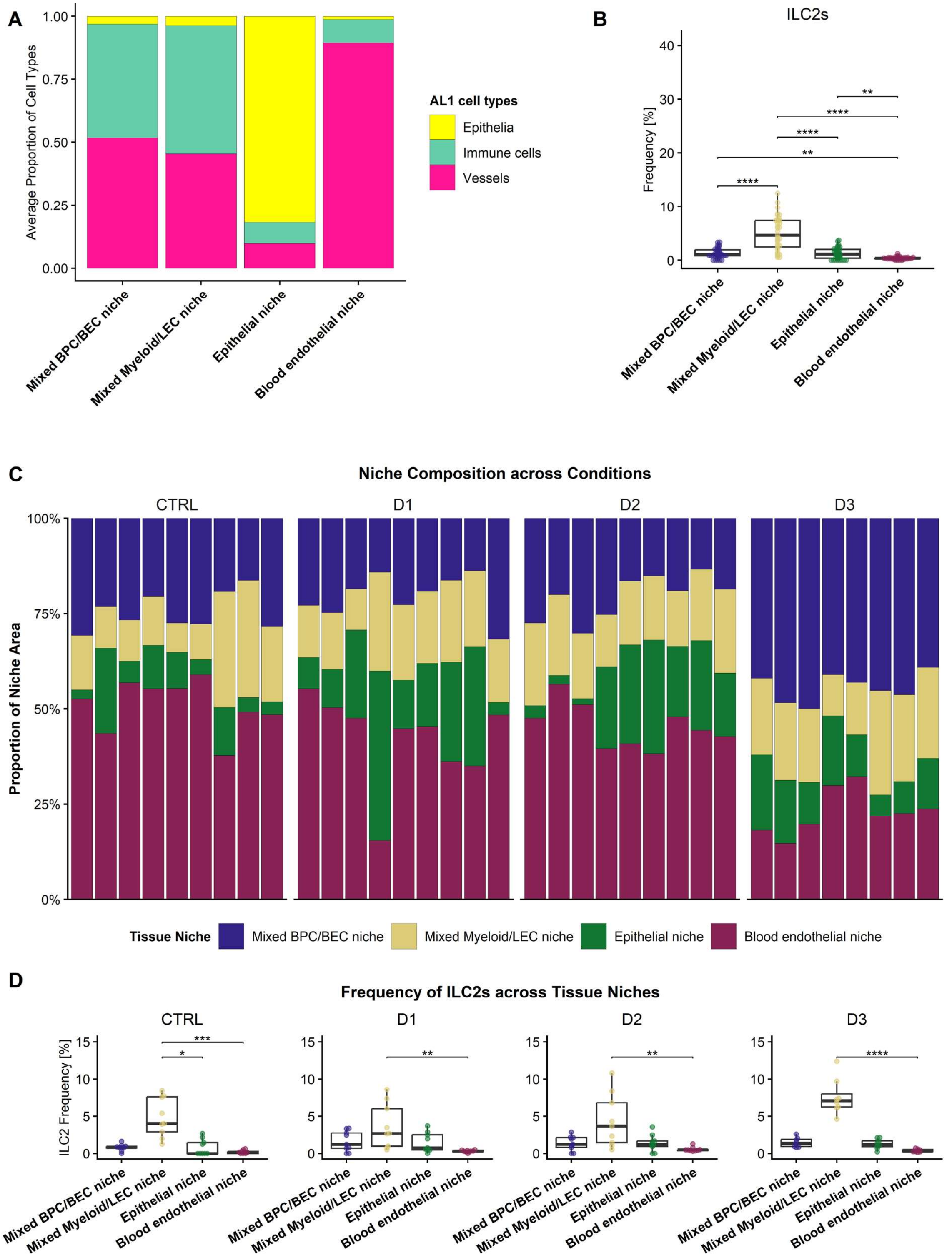

Suppl. Figure 10: Cellular niche composition. (A) Stacked bar plot depicting the identified niches and their composition of AL1 cell types. **(B)** ILC2 frequency across tissue niches. Each dot represents one acquired FOV. For statistical analysis, Kruskal-Wallis-test was used to check for significance between tested groups and effect size, Dunn's test was used as post-hoc test for pairwise comparison. Asterisk marks significance level. N of analyzed FOVs: 35. FOV: fields of view. **(C)** Stacked bar plot depicting the niche abundance per acquired FOV. Each bar represents one acquired FOV. FOV: field of view. **(D)** ILC2 frequency across tissue niches and conditions. Each dot represents one acquired FOV. For statistical

analysis, Kruskal-Wallis-test was used to check for significance between tested groups and effect size, Dunn’s test was used as post-hoc test for pairwise comparison. Asterisk marks significance level. N of analyzed FOVs: 9 for CTRL, D1, and D2; 8 for D3. FOV: fields of view.

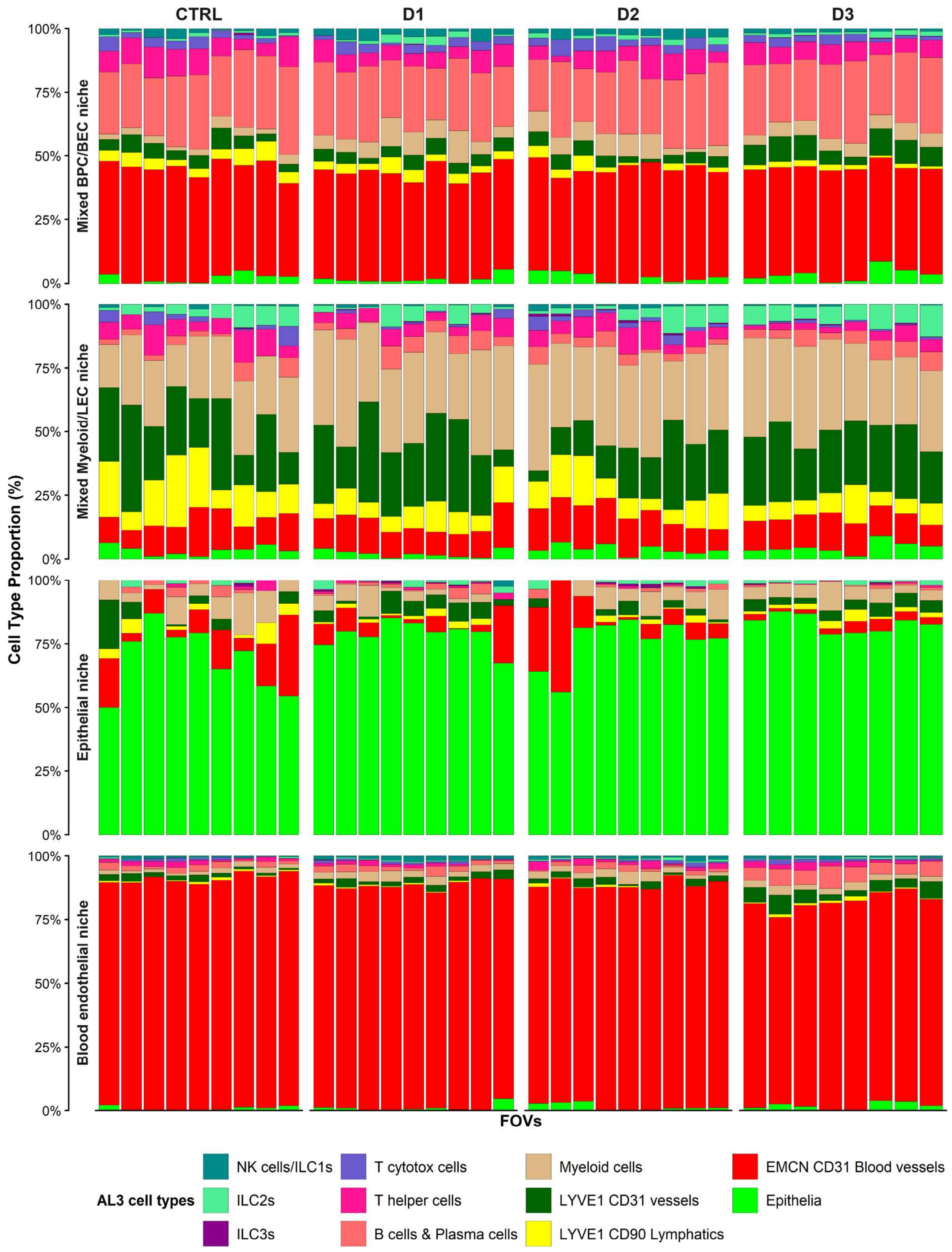

Suppl. Figure 11: Cellular composition of AL3 cell types across niches and conditions. Stacked bar plot depicting the identified niches and their composition of AL3 cell types for each condition. Each bar represents one acquired FOV. FOV: field of view.

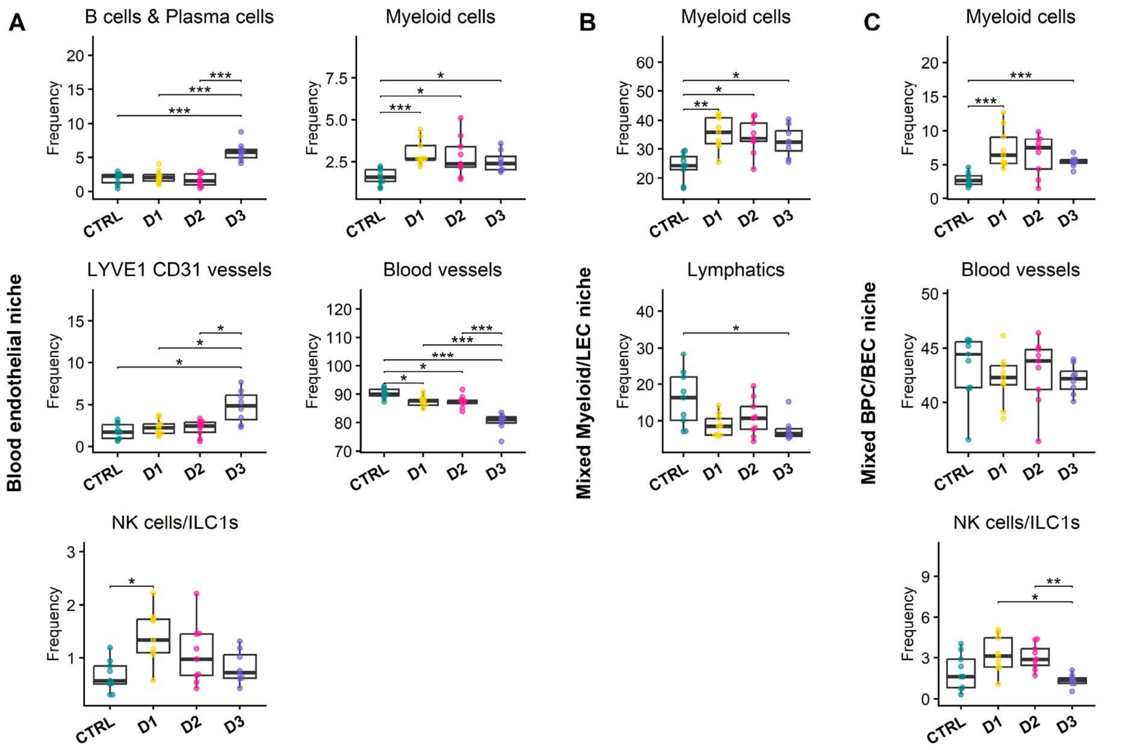

Suppl. Figure 12: Cellular composition within Mixed Myeloid/LEC niche and Blood endothelial niche across conditions. (A) Cellular abundance of B cells & Plasma cells, myeloid cells, NK cells/ILC1s, blood vessels, and LYVE1 CD31 vessels within the blood endothelial niche across conditions. **(B)** Cellular abundance of myeloid cells and lymphatics within the mixed myeloid/LEC niche across conditions. **(C)** Cellular abundance of myeloid cells, blood vessels, and NK cells/ILC1s within the Mixed BPC/BEC niche across conditions. (A-C) Each dot represents one acquired FOV. For statistical analysis, Kruskal-Wallis-test was used to check for significance between tested groups and effect size, Dunn's test was used as post-hoc test for pairwise comparison. Asterisk marks significance level. N of analyzed FOVs: 9 for CTRL, D1, and D2; 8 for D3. FOV: fields of view.

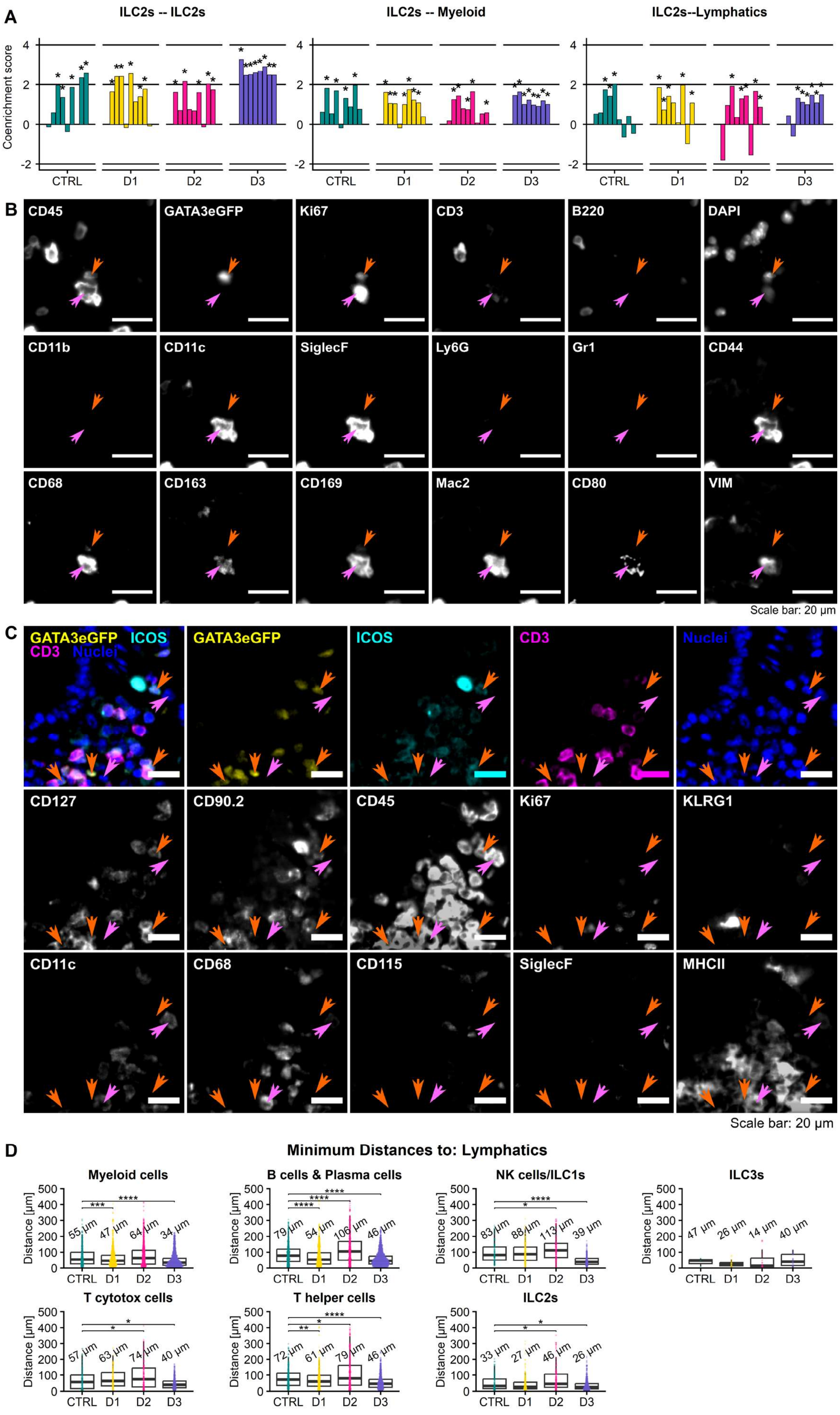
A
ILC2s -- ILC2s
ILC2s -- Myeloid
ILC2s--Lymphatics
Coenrichment score
CTRL
D1
D2
D3
B
CD45
GATA3eGFP
Ki67
CD3
B220
DAPI
CD11b
CD11c
SiglecF
Ly6G
Gr1
CD44
CD68
CD163
CD169
Mac2
CD80
VIM
Scale bar: 20 μm
C
GATA3eGFP ICOS CD3 Nuclei
GATA3eGFP
ICOS
CD3
Nuclei
CD127
CD90.2
CD45
Ki67
KLRG1
CD11c
CD68
CD115
SiglecF
MHCII
Scale bar: 20 μm
D
Minimum Distances to: Lymphatics
Myeloid cells
55 μm 47 μm 64 μm 34 μm
B cells & Plasma cells
79 μm 54 μm 106 μm 46 μm
NK cells/ILC1s
83 μm 88 μm 113 μm 39 μm
ILC3s
47 μm 26 μm 14 μm 40 μm
T cytotox cells
57 μm 63 μm 74 μm 40 μm
T helper cells
72 μm 61 μm 79 μm 46 μm
ILC2s
33 μm 27 μm 46 μm 26 μm
Distance [μm]

Suppl. Figure 13: Coenrichment analysis of ILC2s. (A) Bar plot showing the results of the coenrichment analysis of ILC2s, myeloid cells, and lymphatics with ILC2s across conditions. Each bar represents one analyzed FOV. Asterisks mark p-value lower than 0.05. **(B)** High resolution of zoomed-in exemplary ROI showing identified GATA3eGFP+ CD127+ CD90.2+ LIN-ILC2 (Orange arrow heads) in direct contact with a $CD45^+$ $CD11c^+$ $SiglecF^+$ $CD68^+$ $CD44^+$ activated alveolar macrophage (Pink arrowhead). Arrow heads marking ILC2 (Orange) and activated alveolar macrophage (Pink) are superimposed on IF greyscale images of diverse myeloid markers. **(C)** High resolution of zoomed-in exemplary ROI showing identified $GATA3eGFP^+$ $CD127^+$ $CD90.2^+$ LIN^- ILC2 (Orange arrow heads) co-expressing ICOS and/or MHCII, and/or KLRG1 and are in direct contact with $CD45^+$ $CD11c^+$ $CD68^{+/-}$ myeloid cell (Pink arrowhead). Arrow heads marking ILC2 (Orange) and myeloid cells (Pink) are superimposed on IF overlay and single marker images of diverse markers. **(D)** Box plots depicting the minimal distances of the identified immune cell types to lymphatics as reference cells across conditions. Number represents the median minimum distance for the respective cell type in µm.

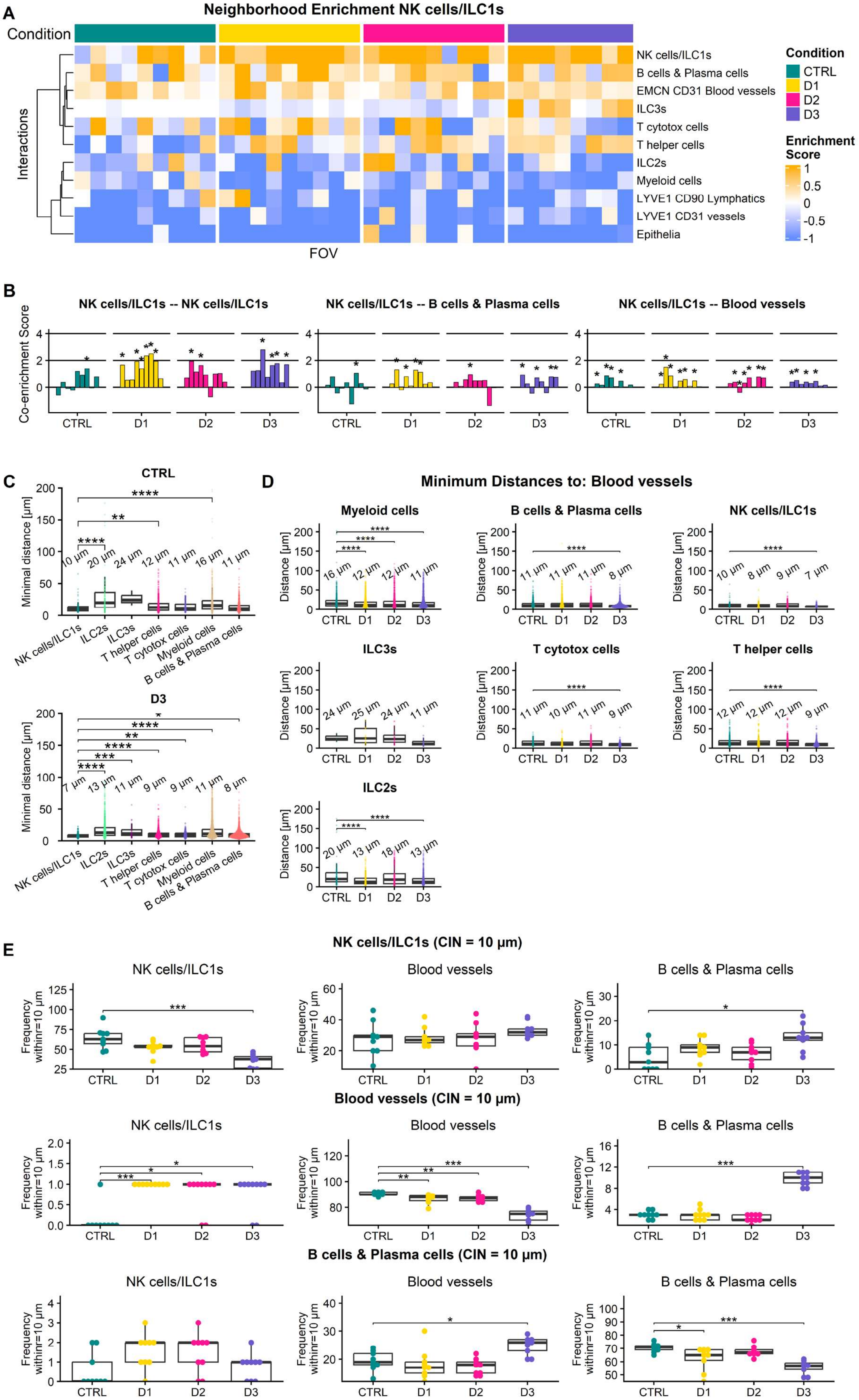
A
Neighborhood Enrichment NK cells/ILC1s
Condition
Interactions
FOV
NK cells/ILC1s
B cells & Plasma cells
EMCN CD31 Blood vessels
ILC3s
T cytotox cells
T helper cells
ILC2s
Myeloid cells
LYVE1 CD90 Lymphatics
LYVE1 CD31 vessels
Epithelia
Condition
CTRL
D1
D2
D3
Enrichment Score
1
0.5
0
-0.5
-1
B
NK cells/ILC1s -- NK cells/ILC1s
NK cells/ILC1s -- B cells & Plasma cells
NK cells/ILC1s -- Blood vessels
Co-enrichment Score
CTRL
D1
D2
D3
C
CTRL
D3
Minimal distance [µm]
NK cells/ILC1s
ILC2s
ILC3s
T helper cells
T cytotox cells
Myeloid cells
B cells & Plasma cells
D
Minimum Distances to: Blood vessels
Myeloid cells
B cells & Plasma cells
NK cells/ILC1s
ILC3s
T cytotox cells
T helper cells
ILC2s
Distance [µm]
E
NK cells/ILC1s (CIN = 10 µm)
Blood vessels (CIN = 10 µm)
B cells & Plasma cells (CIN = 10 µm)
NK cells/ILC1s
Blood vessels
B cells & Plasma cells
Frequency withinr=10 µm

Suppl. Figure 14: NK cells/ILC1s localize in niches with B cells & plasma cells and blood vessels. (A) Heatmap showing the results of the neighborhood coenrichment analysis of NK cells/ILC1s with all annotated cell types from AL3 across analyzed conditions as z-scores. Each column represents one analyzed FOV. **(B)** Bar plot showing the results of the coenrichment analysis of NK cells/ILC1s, B cells & plasma cells, and blood vessels with NK cells/ILC1s across conditions. Each bar represents one analyzed FOV. Asterisks mark p-value lower than 0.05. **(C)** Box plots depicting the minimal distances of the identified immune cell types to blood vessels as reference cells under healthy conditions (Top), and D3 (Bottom). Each dot represents one cell. Number represents the median minimum distance for the respective cell type in µm. **(D)** Box plots depicting the minimal distances of the identified immune cell types to blood vessels as reference cells across conditions. Each dot represents one acquired FOV. Number represents the median minimum distance for the respective cell type in µm. **(E)** Box plots showing the result of the CIN analysis with the frequency of NK cells/ILC1s, B cells & plasma cells, and blood vessels in a 10 µm radius around NK cells/ILC1s, B cells & plasma cells, and blood vessels used as reference cells. Each dot represents one acquired FOV. (D-E) For statistical analysis, Kruskal-Wallis-test was used to check for significance between tested groups and effect size, Dunn's test was used as post-hoc test for pairwise comparison. Asterisk marks significance level. N of analyzed FOVs: 9 for CTRL, D1, and D2; 8 for D3. FOV: fields of view.
